# Supplementary material for: Immunogenicity and safety of measles-mumps-rubella vaccine delivered by the aerosol, intradermal and intramuscular routes in previously vaccinated young adults: a randomized controlled trial protocol
Source: PLoS One. 2025 Mar 21;20(3):e0318893. doi: 10.1371/journal.pone.0318893 (PMC11927902; doi:10.1371/journal.pone.0318893)
Supplement: S2 File — (PDF) [file pone.0318893.s002.pdf]

Introduction

MAXXED Study
Measuring and boosting waning immunity to measles in young adults
Randomised controlled clinical trial - option to register interest at the end of this page

For students needing to receive MMR booster

You can enrol in this research study if you need to receive the MMR booster vaccination as part of your course entry requirements.

Alternative methods of MMR vaccine delivery

If you enrol in the study, you will be randomly assigned one of three vaccine delivery methods when you receive your vaccination at Student Health:

Standard intramuscular delivery (current, standard method, with a standard needle) OR Intradermal vaccine delivery (modified injection to upper layers of skin using a special microneedle device) OR Aerosol delivery (an aerosol vaccine will be created with the Aerogen nebuliser, with the vaccine breathed in via a single-use mouthpiece)

Importance

Waning immunity is a significant concern in countries such as Aotearoa, New Zealand. During the time since your childhood MMR vaccinations, a drop in protective antibodies increases vulnerability to measles infection.

In 2019, an imported measles case led to an outbreak of more than 2,000 cases in Auckland including symptomatic, transmissible, infections in numerous fully immunised young adults, some of them health care professionals.

In 2022, almost 20% of Health Science students entering professional courses at the University of Otago who received a third (booster) dose of MMR vaccine did not achieve measles antibodies above the cut-off level after this extra dose, so there is a need to look for better ways to achieve optimum antibody responses.

Aims of the study

We will investigate whether alternative ways of giving MMR vaccine can give stronger and more lasting protection than standard intramuscular injection.

We will assess if these new methods of delivery are well tolerated and more effective at generating strong and lasting protective measles antibody production.

Follow-up study visits

You'll need to be available to attend three short study visits in the month after vaccination to provide a total of 2 small blood samples and 3 oral fluid swab samples. This is so we can measure and track the development of antibodies. We would also like to ask you for one final blood sample at 12 months after MMR vaccination.

Reimbursement

We will send you a $25 Flexi eGift voucher to your nominated email address for each study visit you attend.

These vouchers can be redeemed at a variety of retailers, letting you choose where to spend them. If all study visits are attended, the total payment you receive will be $125. More information about where these vouchers can be spent can be found at the GiftPay NZ website.

The study will also fund the follow-up lab test that you will be asked to take as part of your course entry screening process.

More information

If you would like to register your interest, clicking on the button and the end of this form will enable you to enter your details so that our study team can contact you. This doesn't immediately enrol you in the study - first we will talk with you and ensure you have all the information you need to decide to enrol, most likely by a zoom chat, but

in-person if you'd prefer.

If you'd like any additional information prior to registering your interest, you can email:

maxxed.study@otago.ac.nz

The study and related advertising materials have been ethically approved by the Health and Disability Ethics Committee (HDEC). Ethics Ref: HDEC 2022 FULL13681

Principal Investigator Study Coordinator
Professor Peter McIntyre

Women's and Children's Health

Dunedin School of Medicine

University of Otago

Dunedin

Tel: 021 2814242

peter.mcintyre@otago.ac.nz

Melanie Millier

Women's and Children's Health

Dunedin School of Medicine

University of Otago

Dunedin

melanie.millier@otago.ac.nz

Click here to see the "at a glance" table relating to the study

◯ Study participation at a glance

Study participation at a glance -

Visits highlighted in gold are solely study-related and not part of the existing screening and immunisation procedures

Screening blood test Pre-enrolment talk to study team Vaccination at Student Health e-diary Post-MMR vaccination study visits
Screening results via Student Health: Insufficient antibodies to Measles or Mumps {~15 min} Visit # 1

{~1 hour}

Electronic entries of temperature and any side effects Visit #2 {~45 min} Visit #3 {~45 min} Visit #4 {~45 min} Visit #5 {~30 min}
Randomly assigned to one of three MMR vaccine delivery routes

Standard IM

OR

Microneedle (Intradermal)

OR

Aerosol

3-5 days after MMR

6-8 days after MMR 13-15 days after MMR 28-40 days after MMR ~12 months after MMR
Oral Swab Oral Swab Oral Swab
Blood sample Blood sample Blood sample

Use of remaining serum from SCL labs Use of remaining serum from SCL labs
$25 Flexi Gift voucher $25 Flexi Gift voucher $25 Flexi Gift voucher $25 Flexi Gift voucher Study pays for Measles blood test $25 Flexi Gift voucher

---

You can watch a brief video here - less than 2 min

Link to full participant information sheet - 13 pages, but you don't need to look at it now

Please note, it is not necessary that this be completed ahead of talking to our study team. We will go through the information and consent process with you once you provide your contact details

[Attachment: "Participant information and consent form_MMR clinical trial.pdf"]

Would you like to register your interest in participating in the MAXXED MMR clinical trial study?*
Please select your option below. To complete your registration you will also need to continue to the next page to leave us your details
(* Indicating that you're interested will mean we will contact you to explain how the study works and answer any questions you might have to help decide if you'd like to enrol.)

◯ Yes, I am interested in finding out more
◯ No thanks, I am not interested in participating in this research study

Thanks for you interest, please let us know your contact details

Today's date	______________________
First name	______________________ (First name)
Preferred name	______________________ (If different from first name)
Surname	______________________
[first_names] [surname]	______________________
Student email address	______________________ (Please enter your Otago University email address)
Preferred email address for our study team to contact you at	______________________
Mobile phone number	______________________ (Please let us know the best phone number to reach you on)
Txt detail -@mtxt.co.nz	______________________
MultiTXT email	______________________
mtxt destination email	______________________
What is your date of birth	______________________
Age	______________________
Current age in years (automatically calculated and rounded up or down to nearest year)	______________________

Once we have your details we'll send you an email and a text about scheduling a pre-enrolment meeting

We're planning to hold these meetings via zoom, please indicate if this is ok	◯ Yes zoom is fine<br>◯ I would prefer to meet face-to-face<br>(If you like, you can bring another friend or family member)
Please let us know any other information that may help in scheduling a pre-enrolment meeting with you	__________________________________<br>(Any details such as a preferred day / time to schedule a meeting (approx 10 - 20min) ahead of your vaccination clinic)

Provisional Consent

Please complete the provisional consent form

Date

____

Electronic Provisional Consent Form
Measuring and boosting waning immunity to measles in young adults

Randomised Controlled Clinical Trial

______

I have read the Participant Information Sheet and understand the aims of this research project.

______ I have had sufficient time to talk with people of my choice about participating in the study.

______ I confirm that I meet the criteria for participation explained in the Information Sheet.

______ All my questions about the project have been answered to my satisfaction, and I understand that I am free to request further information at any stage.

______ I know that my participation in the project is entirely voluntary, and that I am free to withdraw from the project at any time without disadvantage.

______ I understand the risks of discomfort or harm explained in the Information Sheet.

______ I agree to donate my blood and oral fluid samples for analysis in this study

______ I understand that my blood samples will be sent to an overseas laboratory for analysis and will be kept there for up to 5 years.

______ I know that when the project is completed all personal identifying information will be removed from the paper records and electronic files which represent the data from the project, and that these will be placed in secure storage and kept for at least ten years.

______ I understand that the results of the project may be published and be available in the University of Otago Library, but that any personal identifying information will remain confidential, and will not appear in any spoken or written report of the study.

______ I understand the arrangements for payment in compensation for time and inconvenience due to my participation in this study, and have had my questions about this addressed to my satisfaction.

______ I am willing to be contacted for further research

Your first name

____

Your surname name	________________
Your date of birth	________________
Ethnicity	☐ New Zealand European<br>☐ Mâori<br>☐ Samoan<br>☐ Cook Island Mâori<br>☐ Tongan<br>☐ Niuean<br>☐ Chinese<br>☐ Indian<br>☐ Other such as Dutch, Japanese, Tokelauan<br>(Select ALL that apply)
Gender	◯ Male<br>◯ Female<br>◯ Non binary
As you have identified your ethnicity as 'other' please state your ethnicity.	________________
As you have selected your ethnicity as Mâori, please identify your iwi	________________
Your signature	____________________

Area for participant to complete.

Participants Full Name ______

Participants signature ______

Date that participant signed Consent form ______

Area for researcher to complete.

Researcher's Full Name ______

Researcher's signature ______

Date that researcher signed Consent form ______

Emergency Contact

Record ID __________________________________

Person to contact in case of emergency

Full name

____

Contact persons relationship to you?

____

Emergency contact phone number

____

Local Lab Screening Serology Data

Please complete the survey below.

Thank you!

Screening data from Lab Serology

First name(s) __________________________________

Surname __________________________________

Date of Birth __________________________________

Details of other testing lab __________________________________

Screening Lab Data
Date of test ______ Testing lab ______
Lab test ID# ______ Diagnostic test used ______

Measles (morbilli) IgG comment ______ Measles (morbilli) IgG titre (AU/mL) ______
Mumps IgG comment ______ Mumps IgG titre (AU/mL) ______
Rubella IgG comment ______ Rubella IgG titre (AU/mL) ______

Aliquot of serum stored for use in the study
◯ yes
◯ no

Aliquot location (temporary) __________________________________

Comments ____

Country of birth and Measles survey

Kia ora [first_names],

To help with the analysis of data within the MAXXED study, we would like to ask you to complete a short survey relating to your country of birth and any previous measles infection and/or immunisations.

This will enhance the quality of the data you have already contributed to during your study participation.

Warm regards,

MAXXED study team

In which country were you born:	◯ I was born in Aotearoa, New Zealand, and have always lived here<br>◯ I was born in Aotearoa, New Zealand, but have also lived in another country (or countries)<br>◯ I was born in a country other than Aotearoa, New Zealand
Please record your country of birth	__________________________________
What year did you arrive to live in Aotearoa, NZ?	__________________________________
Have you also lived in other countries other than your birth country and Aotearoa, New Zealand?	◯ Yes<br>◯ No<br>(Please only consider countries you have lived in, rather than countries you have visited)

Please list the countries you have lived - one per row, including the year you arrived, and the year you left
Country Year of arrival Year of departure - enter the year

OR type "current" if entering data for your current country of residence (NZ)

______ ______ until ______

______ ______ until ______
______ ______ until ______

Measles infection

Have you ever experienced a measles infection?	◯ Yes<br>◯ No

What year (approximately) did this infection occur? ______ Was the measles infection confirmed by either a medical diagnosis or a postive lab test? ______

Measles Vaccinations

Please select the option describing your Measles vaccination history before enetering your current course of study	◯ I received ONE dose of MMR (or other measles vaccination) during my childhood<br>◯ I received TWO doses of MMR (or other measles vaccination) during my childhood<br>◯ I DID NOT receive any doses of MMR (or other measles vaccination) during my childhood<br>◯ I am unsure
Do you recall whether your MMR vaccination record was provided to Student Health, or was a declaration of vaccination provided?	◯ I provided my vaccination records to Student Health<br>◯ I provided a declaration that I received MMR vaccination as I was unable to locate the written record<br>◯ I am not sure

Visit 1a Study Nurse

[first_names] ([preferred_name]) [surname]	
Research Nurse name	◯ Emma Collins<br>◯ Elizabeth Cook<br>◯ Louise Fletcher<br>◯ Amanda Inglis-Flaws<br>◯ Melanie Millier<br>◯ other<br>(Name of study staff member taking the screening info)
Staff name	______________________________
Date of screening	______________________________
The participant has given consent to participate in the study	◯ Yes ◯ No<br>(Must be yes to continue with the study vaccination)
The subject has received a copy of the signed consent form via email	◯ Yes ◯ No<br>(This will be sent after being scanned)
Please upload a pdf copy of the completed consent form	

Exclusion Criteria

- ☐ Acute illness within 5 days of planned study vaccination ______
- ☐ Proven anaphylaxis to MMR vaccine or vaccine component ______
- ☐ Significantly immunocompromised ______
- ☐ Received a live vaccine within four weeks of planned study vaccination, incl BCG or Varicella ______
- ☐ Intravenous immunoglobulin or blood transfusion within preceding 11 months ______
- ☐ Pregnancy ______

One or more exclusion criteria have been selected. Confirm exclusion criteria and discontinue study participation?	◯ Yes ◯ No<br>(Continued study participation is not possible once confirmed)
The participant is willing and able to adhere to the study protocol (If No, exclude).	◯ Yes ◯ No

Inhaled Substances

Does the participant currently, or have they previously smoked cigarettes or inhaled vape products?	◯ Yes<br>◯ No

Cigarette Use

	Never	Occasionally (up to once per month)	Less than once per week	Once per week	Between 2 and 6 times per week	Once daily	More than once daily
Cigarette Smoking - average use	○	○	○	○	○	○	○

Vaping product use

	Never	Occasionally (up to once per month)	Less than once per week	Once per week	Between 2 and 6 times per week	Once daily	More than once daily
Inhaled vaping products - average use	○	○	○	○	○	○	○

Physical Observations

Height in cm

____
(Height in cm)

Weight in kg

____
(Weight in Kg)

BMI

____

Temperature

____
(Record to 1 decimal place)

Heart rate / Pulse

____
(BPM)

Medical history

Does the participant have any ongoing medical conditions?

○ Yes
○ No

Significant Medical History requiring regular follow-up

Condition	Ongoing?
______	______
______	______
______	______
______	______

Upload document detailing subjects significant medical history - optional

Medications Including all prescription and non-prescription medications

Does the participant regularly take any prescription medication?

◯ Yes
◯ No
(Including insulin, contraceptives, asthma medication etc)

Medication Is medication ongoing Frequency

Medication	Is medication ongoing	Frequency
______	______	______
______	______	______
______	______	______
______	______	______

Source document for medications added to subject's file - optional

Does the participant regularly take non-prescription medications or supplements?

◯ Yes
◯ No

Medication/supplement Frequency

Medication/supplement	Frequency
______	______
______	______
______	______
______	______
______	______

Allergies

Does the participant have any allergies, including food, medication or contact allergies?

◯ Yes
◯ No

Medication / Vaccine / Food / Tape etc Effect Date it first occurred / diagnosed

Medication / Vaccine / Food / Tape etc	Effect	Date it first occurred / diagnosed
______	______	______
______	______	______
______	______	______

Visit 1b Randomization

Please complete the survey below.

Thank you!

General Comments

Randomization Group

- ◯ Intramuscular
- ◯ Intra dermal
- ◯ Aerosol

Visit 1c Vaccination Form

Please complete the survey below.

Thank you!

Final check before Vaccination

[first_names] [surname]

Date of birth [dob_d66641]

Please verify the participant has signed the consent.	◯ Yes<br>◯ No
Has the participant received any vaccinations within 28 days prior to planned study vaccination?	◯ Yes<br>◯ No
Vaccination received	☐ Hepatitis B<br>☐ Varicella<br>☐ Pertussis<br>☐ Other
As you have identified in the prior vaccination question that you have had "Other" vaccination/s please document what those other vaccinations are?	__________________________________

Screening blood sample stored at SCL for use in the study according to lab screening data entry

NO screening blood sample has been kept for use in this study according to the screening lab data entry.

A blood sample will need to be taken prior to vaccination

Participant is aware they need to provide a pre-vaccination blood sample, and agree to this	◯ yes<br>◯ no

Vaccination

Date of vaccination ______ Time of vaccination ______
Name of study nurse administering vaccine ______ ______
Randomly assigned delivery method: [randomization_group]
MMR delivery method given ______
Please comment if the assigned method is not the method given ______
If needle delivery, was this delivered to the left or right arm? ______
MMR vaccine batch # ______

Vaccination given	◯ Yes<br>◯ No
Were any issues/problems identified during 30 minute post administration observation period?	◯ Yes<br>◯ No

Post vaccination issues identified.	☐ Increased heart rate<br>☐ Reported breathing difficulty<br>☐ Reported pain at injection site<br>☐ Reported rash<br>☐ Feeling faint<br>☐ Mouth tingling<br>☐ Other
Please specify what the other issues are?	__________________________________
Following the reported issues identified post vaccination. Please document what actions and follow up were undertaken.	____

12 Hour post MMR e-diary

randomised to ______________________________

e-diary 12 hour post MMR check

Date of Check :- ______

Time of Check :- ______

e-diary post vaccination reaction check

Since you received the [random_check] delivery option for MMR vaccine

Have you experienced any of the following side-effects from your vaccination? ______

Vomiting Please identify the severity of your symptom ______

Diarrhoea Please identify the severity of your symptom ______

Headaches Please identify the severity of your headache symptom ______

Fatigue/ tiredness? Please identify the severity of your fatigue/ tiredness symptom ______

New or worsening rash Please identify the severity of your new or worsening rash symptom ______

Muscle or joint pain which is either new or worsening. Please identify the severity of your Muscle or joint pain which either new or worsening ______

Have you taken any medication to alleviate symptoms of muscle or joint pain in the last 12 hours?______

Please list the medication you have taken to alleviate the muscle or joint pain in the past 12 hours? ______________________________

Please let us know where on your body you are experiencing this rash ______________________________

If you would like to, you can choose the option to upload an image of your rash.

(It can be helpful to include a tape measure in the photograph to help assess the size of the rash)

Fever

Please indicate if you are able to take your oral temperature using the digital thermometer provided	◯ Yes I can do this<br>◯ Sorry, this is not possible for me to do
Please choose the temperature range of your oral temperature reading	◯ Up to, and including, 37.9 degrees Celcius<br>◯ 38.0 degrees Celcius or above
Please enter your your oral temperature, in degrees Celcius (to 1 decimal place)	________________________________
Your temperature is recorded as 38.0 deg Celcius or above. Please confirm this is correct.	◯ Yes<br>(If you need to correct the value you entered you can reset your entered temperature in the previous entry field)
Please identify the severity of your fever?	◯ ≥38.0 degC up to and including 38.4 degC<br>◯ from 38.5 degC up to and including 38.9 degC<br>◯ from 39.0 degC up to and including 40.0 degC<br>◯ Above 40.0 degC<br>(After measuring an oral temperature reading, please classify your temperature in the applicable range)
In the last 12 hour period, what was the highest temperature that you recorded?	________________________________<br>(degrees centigrade)
Have you taken any medication to alleviate symptoms of a fever in the last 12 hours?	◯ Yes<br>◯ No
Have you experienced chills or shivering with your fever	◯ Yes<br>◯ No

Reaction site symptoms

Reaction site symptoms Since you received an [random_check] vaccination in your [left_right] arm, please answer the following questions with this site in mind:

Have you had redness at the MMR injection site? ______
Have you had swelling at the MMR injection site? ______
Have you had pain at the MMR injection site? ______

Please select the options that best describes the maximum dimension of redness at your injection site	◯ >2.0 cm to 5.0 cm<br>◯ >5.0 cm to 10.0 cm<br>◯ >10 cm
Are you experiencing any weeping (pus) at the injection site?	◯ Yes<br>◯ No
You can choose to upload a photo of your injection site redness here	(Please take and upload an photo of the site - if possible please include a tape measure beside the area of concern)
Please identify the severity of the swelling at your injection site?	◯ >2.0 cm to 5.0 cm<br>◯ >5.0 cm to 10.0 cm<br>◯ >10 cm
You can choose to upload a photo of your injection site swelling here	(It may be helpful to also include a tape measure in the photo so that we can assess the size of the swollen area)
Please identify the severity of your injection site pain	◯ Does not interfere with activity<br>◯ Interferes with activity<br>◯ Prevents daily activity<br>◯ GP visit or Emergency Department visit
Have you taken any medication to alleviate symptoms of pain in the last 12 hours?	◯ Yes<br>◯ No
Please list the medication you have taken to alleviate any symptoms of pain in the past 12 hours	__________________________________
Please comment if you have noticed any other symptoms	____

24 Hour post MMR e-diary

randomised to ________________________________

e-diary 24 hour post MMR check

Date of Check :- ______

Time of Check :- ______

e-diary 24 hour post vaccination reaction check

In the last 12 hours or so since your first 12 hour e-diary entry, have you experienced any of the following side-effects from the [random_check] MMR vaccine?

Have you experienced any of the following side-effects from your vaccination? ______

Vomiting Please identify the severity of your symptom ______

Diarrhoea Please identify the severity of your symptom ______

Headaches Please identify the severity of your headache symptom ______

Fatigue/ tiredness? Please identify the severity of your fatigue/ tiredness symptom ______

New or worsening rash Please identify the severity of your new or worsening rash symptom ______

Muscle or joint pain which is either new or worsening. Please identify the severity of your Muscle or joint pain which either new or worsening ______

Have you taken any medication to alleviate symptoms of muscle or joint pain in the last 12 hours?______

Please list the medication you have taken to alleviate the muscle or joint pain in the past 12 hours? ________________________________

Please let us know where on your body you are experiencing this rash ________________________________

If you would like to, you can choose the option to upload an image of your rash.

(It can be helpful to include a tape measure in the photograph to help assess the size of the rash)

Fever	
Please indicate if you are able to take your oral temperature using the digital thermometer provided	◯ Yes I can do this<br>◯ Sorry, this is not possible for me to do
Please choose the temperature range of your oral temperature reading	◯ Up to, and including, 37.9 degrees Celcius<br>◯ 38.0 degrees Celcius or above
Please enter your your oral temperature, in degrees Celcius (to 1 decimal place)	________________________
Your temperature is recorded as 38.0 deg Celcius or above. Please confirm this is correct.	◯ Yes<br>(If you need to correct the value you entered you can reset your entered temperature in the previous entry field)
Please identify the severity of your fever?	◯ ≥38.0 degC up to and including 38.4 degC<br>◯ from 38.5 degC up to and including 38.9 degC<br>◯ from 39.0 degC up to and including 40.0 degC<br>◯ Above 40.0 degC<br>(After measuring an oral temperature reading, please classify your temperature in the applicable range)
In the last 12 hour period, what was the highest temperature that you recorded?	________________________<br>(Please record in degrees Celcius to 1 decimal place)
Have you taken any medication to alleviate symptoms of a fever in the last 12 hours?	◯ Yes<br>◯ No
Have you experienced chills or shivering with your fever	◯ Yes<br>◯ No

Reaction site symptoms

Reaction site symptoms over the last 12 hours Since your last 12 hour e-diary entry, and with reference to your [random_check] MMR vaccination, please answer the following questions with your [left_right] arm injection site in mind:

Have you had redness at the MMR injection site? ______
Have you had swelling at the MMR injection site? ______
Have you had pain at the MMR injection site? ______

Please select the options that best describes the maximum dimension of redness at your injection site	◯ >2.0 cm to 5.0 cm<br>◯ >5.0 cm to 10.0 cm<br>◯ >10 cm
Are you experiencing any weeping (pus) at the injection site?	◯ Yes<br>◯ No
You can choose to upload a photo of your injection site redness here	(Please take and upload an photo of the site - if possible please include a tape measure beside the area of concern)
Please identify the severity of the swelling at your injection site?	◯ >2.0 cm to 5.0 cm<br>◯ >5.0 cm to 10.0 cm<br>◯ >10 cm
You can choose to upload a photo of your injection site swelling here	(It may be helpful to also include a tape measure in the photo so that we can assess the size of the swollen area)
Please identify the severity of your injection site pain	◯ Does not interfere with activity<br>◯ Interferes with activity<br>◯ Prevents daily activity<br>◯ GP visit or Emergency Department visit
Have you taken any medication to alleviate symptoms of pain in the last 12 hours?	◯ Yes<br>◯ No
Please list the medication you have taken to alleviate any symptoms of pain in the past 12 hours	______________________________
Please comment if you have noticed any other symptoms	______________________________

48 Hour post MMR e-diary

randomised to ________________________________

e-diary 48 hour post MMR check

Date of Check :- ______

Time of Check :- ______

e-diary 48 hour post vaccination reaction check

Over the past 24 hours or so (since your 24 hour e-diary record), and thinking about your [random_check] MMR vaccine, have you experienced any of the following side-effects?

Have you experienced any of the following side-effects from your vaccination? ______

Vomiting Please identify the severity of your symptom ______

Diarrhoea Please identify the severity of your symptom ______

Headaches Please identify the severity of your headache symptom ______

Fatigue/ tiredness? Please identify the severity of your fatigue/ tiredness symptom ______

New or worsening rash Please identify the severity of your new or worsening rash symptom ______

Muscle or joint pain which is either new or worsening. Please identify the severity of your Muscle or joint pain which either new or worsening ______
Have you taken any medication to alleviate symptoms of muscle or joint pain in the last 12 hours?______

Please list the medication you have taken to alleviate the muscle or joint pain in the past 24 hours? ________________________________

Please let us know where on your body you are experiencing this rash ________________________________

If you would like to, you can choose the option to upload an image of your rash.

(It can be helpful to include a tape measure in the photograph to help assess the size of the rash)

Fever

Please indicate if you are able to take your oral temperature using the digital thermometer provided	◯ Yes I can do this<br>◯ Sorry, this is not possible for me to do
Please choose the temperature range of your oral temperature reading	◯ Up to, and including, 37.9 degrees Celcius<br>◯ 38.0 degrees Celcius or above
Please enter your your oral temperature, in degrees Celcius (to 1 decimal place)	__________________________________
Your temperature is recorded as 38.0 deg Celcius or above. Please confirm this is correct.	◯ Yes<br>(If you need to correct the value you entered you can reset your entered temperature in the previous entry field)
Please identify the severity of your fever?	◯ ≥38.0 degC up to and including 38.4 degC<br>◯ from 38.5 degC up to and including 38.9 degC<br>◯ from 39.0 degC up to and including 40.0 degC<br>◯ Above 40.0 degC<br>(After measuring an oral temperature reading, please classify your temperature in the applicable range)
In the last 24 hour period, what was the highest temperature that you recorded?	__________________________________<br>(please record in degrees Celcius to 1 decimal place)
Have you taken any medication to alleviate symptoms of a fever in the last 24 hours?	◯ Yes<br>◯ No
Have you experienced chills or shivering with your fever	◯ Yes<br>◯ No

Reaction site symptoms

Reaction site symptoms over the past 24 hours Over the last 24 hours or so since your last e-diary entry, and thinking about the [random_check] MMR vaccination in your [left_right] arm, please answer the following questions with this site in mind:

Have you had redness at the MMR injection site? ______
Have you had swelling at the MMR injection site? ______
Have you had pain at the MMR injection site? ______

Please select the options that best describes the maximum dimension of redness at your injection site	◯ >2.0 cm to 5.0 cm<br>◯ >5.0 cm to 10.0 cm<br>◯ >10 cm
Are you experiencing any weeping (pus) at the injection site?	◯ Yes<br>◯ No
You can choose to upload a photo of your injection site redness here	(Please take and upload an photo of the site - if possible please include a tape measure beside the area of concern)
Please identify the severity of the swelling at your injection site?	◯ >2.0 cm to 5.0 cm<br>◯ >5.0 cm to 10.0 cm<br>◯ >10 cm
You can choose to upload a photo of your injection site swelling here	(It may be helpful to also include a tape measure in the photo so that we can assess the size of the swollen area)
Please identify the severity of your injection site pain	◯ Does not interfere with activity<br>◯ Interferes with activity<br>◯ Prevents daily activity<br>◯ GP visit or Emergency Department visit
Have you taken any medication to alleviate symptoms of pain in the last 24 hours?	◯ Yes<br>◯ No
Please list the medication you have taken to alleviate any symptoms of pain in the past 24 hours	__________________________________
Please comment if you have noticed any other symptoms	____

Visit 2 Study Visit @ Day 3-5 post MMR

[first_names] [surname] Date of Birth [dob_d66641] (preferred name:[preferred_name]) Method of delivery [random_check]

Visit 2: Day 3-5 post MMR

Date of Check :- ______

Time of Check :- ______

Check for any unreported adverse events via e diary review ______

During this visit, are there any lasting side-effects following vaccination? ______

Vomiting Please identify the severity of the vomiting ______

Diarrhoea Please identify the severity of the diarrhoea ______

Headaches Please identify the severity of the headache symptoms ______

Fatigue/ tiredness? Please identify the severity of fatigue/ tiredness symptom ______

New or worsening rash Please identify the severity of the new or worsening rash symptom ______
Optional image upload: rash ______

New or worsening muscle or joint pain Please identify the severity of the new or worsening symptoms ______

Has [first_names] taken any medication to alleviate symptoms of muscle or joint pain in the last 12 hours?______
Please list the medication(s) taken to alleviate the muscle or joint pain in the past 12 hours? ______

Fever

If the participant feels feverish or unwell, please take an oral temperature reading, and record to 1 decimal place (deg Celcius)	______________________________
Please grade the severity of the fever	◯ ≥38.0 degC up to and including 38.4 degC<br>◯ from 38.5 degC up to and including 38.9 degC<br>◯ from 39.0 degC up to and including 40.0 degC<br>◯ Above 40.0 degC<br>(After measuring an oral temperature reading, please classify your temperature in the applicable range)

Has any antipyretic medication for fever been taken in the last 12 hours? ______
Which fever (antipyretic) medication was taken? ______

Do chills/shivering accompany the fever?	◯ Yes<br>◯ No

Local reaction site symptoms

Since MMR vaccination was delivered to the [left_right] arm:

Is any redness present at the injection site? ______ Please identify the severity of redness ______
Optional image upload for 'redness' ______
Is swelling present at the injection site? ______ Please identify the severity of the swelling ______
Optional image upload for 'swelling' ______

Is there pain at the MMR injection site ?
This is recorded as given on the [left_right] ______ Please identify the severity of the injection site pain ______
Has any medication been used to alleviate symptoms of pain at the injection site in the last 12 hours? ______ Please list the medication(s) taken to alleviate any symptoms of pain at the injection site in the past 12 hours ______

Other symptoms

Please comment on any other side effects present

____

Collect an oral fluid sample using the Oracol pro device

Time sample collected: ______
Oral sample tube labelling convention: ______

Next study day appointment visit scheduled for ;- ______

Visit 3 Study Visit @ Day 6-8 post MMR

[first_names] [surname] Date of Birth [dob_d66641] (preferred name:[preferred_name]) Method of delivery [random_check]

Visit 3: Day 6-8 post MMR

Date of Check :- ______

Time of Check :- ______

Check for any unreported adverse events via e diary review ______

Since the last study visit, are there any side-effects possibly related to vaccination? ______

Vomiting Please identify the severity of the vomiting ______

Diarrhoea Please identify the severity of the diarrhoea ______

Headaches Please identify the severity of the headache symptoms ______

Fatigue/ tiredness? Please identify the severity of fatigue/ tiredness symptom ______

New or worsening rash Please identify the severity of the new or worsening rash symptom ______
Optional image upload: rash ______

New or worsening muscle or joint pain Please identify the severity of the new or worsening symptoms ______

Has [first_names] taken any medication to alleviate symptoms of muscle or joint pain in the last 12 hours?______
Please list the medication(s) taken to alleviate the muscle or joint pain in the past 12 hours? ______

Fever

If feeling hot or feverish, please take an oral temperature reading, and record to 1 decimal place (deg Celcius)	__________________________
Please grade the severity of the fever	◯ ≥38.0 degC up to and including 38.4 degC<br>◯ from 38.5 degC up to and including 38.9 degC<br>◯ from 39.0 degC up to and including 40.0 degC<br>◯ Above 40.0 degC<br>(After measuring an oral temperature reading, please classify your temperature in the applicable range)

Has any antipyretic medication for fever been taken in the last 12 hours? ______
Which fever (antipyretic) medication was taken? ______

Do chills/shivering accompany the fever?	◯ Yes<br>◯ No

Local reaction site symptoms

Since MMR vaccination was delivered to the [left_right] arm:

Is any redness present at the injection site? ______ Please identify the severity of redness ______
Optional image upload for 'redness' ______
Is swelling present at the injection site? ______ Please identify the severity of the swelling ______
Optional image upload for 'swelling' ______

Is there pain at the MMR injection site ?
This is recorded as given on the [left_right] ______ Please identify the severity of the injection site pain ______
Has any medication been used to alleviate symptoms of pain at the injection site in the last 12 hours? ______ Please list the medication(s) taken to alleviate any symptoms of pain at the injection site in the past 12 hours ______

Other symptoms

Please comment on any other side effects present

____

Collect an oral fluid sample using the Oracol pro device

Time sample collected: ______
Label convention (oral fluid): ______
Collect a blood sample for serum isolation   Method of blood sampling ______
Time of blood sample collection ______
Label convention (serum): ______

Next Study Visit scheduled for: ______

Visit 4 Study Visit @ Day 13-15 post MMR

[first_names] [surname] Date of Birth [dob_d66641] (preferred name:[preferred_name]) Method of delivery [random_check]

Visit 4: Day 13-15 post MMR

Date of Check :- ______

Time of Check :- ______

Check for any unreported adverse events via e diary review ______

During this visit, are there any lasting side-effects following vaccination? ______

Vomiting Please identify the severity of the vomiting ______

Diarrhoea Please identify the severity of the diarrhoea ______

Headaches Please identify the severity of the headache symptoms ______

Fatigue/ tiredness? Please identify the severity of fatigue/ tiredness symptom ______

New or worsening rash   Please identify the severity of the new or worsening rash symptom ______
Optional image upload: rash ______

New or worsening muscle or joint pain Please identify the severity of the new or worsening symptoms ______

Has [first_names] taken any medication to alleviate symptoms of muscle or joint pain in the last 12 hours?______
Please list the medication(s) taken to alleviate the muscle or joint pain in the past 12 hours? ______

Fever

If the participant feels feverish or unwell, please take an oral temperature reading, and record to 1 decimal place (deg Celcius)	______________________
Please grade the severity of the fever	◯ ≥38.0 degC up to and including 38.4 degC<br>◯ from 38.5 degC up to and including 38.9 degC<br>◯ from 39.0 degC up to and including 40.0 degC<br>◯ Above 40.0 degC<br>(After measuring an oral temperature reading, please classify your temperature in the applicable range)

Has any antipyretic medication for fever been taken in the last 12 hours? ______
Which fever (antipyretic) medication was taken? ______

Do chills/shivering accompany the fever?	◯ Yes<br>◯ No

Local reaction site symptoms

Since MMR vaccination was delivered to the [left_right] arm:

Is any redness present at the injection site? ______ Please identify the severity of redness ______
Optional image upload for 'redness' ______
Is swelling present at the injection site? ______ Please identify the severity of the swelling ______
Optional image upload for 'swelling' ______

Is there pain at the MMR injection site ?
This is recorded as given on the [left_right] ______ Please identify the severity of the injection site pain ______
Has any medication been used to alleviate symptoms of pain at the injection site in the last 12 hours? ______ Please list the medication(s) taken to alleviate any symptoms of pain at the injection site in the past 12 hours ______

Other symptoms

Please comment on any other side effects present

____

Collect an oral fluid sample using the Oracol pro device

Time sample collected: ______
Label convention (oral fluid): ______
Collect a blood sample for serum isolation   Method of blood sampling ______
Time of blood sample collection ______
Label convention (serum): ______

Next Study Visit scheduled for: ______

Visit 5 Labs Serology 28 - 42 Days Post MMR

Please complete the survey below.

Thank you!

Post-vaccination data from Lab Serology

First name ________________________________

Surname ________________________________

Date of Birth ________________________________

Screening Lab Data

Date of test ______

Time since MMR: ______

Testing lab ______ If other lab: ______
Lab test ID# ______

Diagnostic test used ______

Measles (morbilli) IgG result ______

Measles (morbilli) IgG titre (AU/mL) ______

Mumps IgG comment ______

Mumps IgG titre (AU/mL) ______

Rubella IgG comment ______

Rubella IgG titre (AU/mL) ______

Aliquot kept
- ◯ Yes
- ◯ No

Aliquot location (temporary)
______ ______ ______

Notes on post MMR testing ________________________________

Visit 6 @12 Month post MMR

12 months post MMR

Has patient completed the 12 month blood test?
◯ No
◯ Yes

12 month post MMR Lab Serology

Date of Test ________________________

First namelab ________________________

Surname ________________________

Date of Birth ________________________

SCL followup specimen ID ________________________

Measles antibody result ________________________
(AU/ml)

Mumps antibody result ________________________
(AU/ml)

Completion and withdrawl

Study Completion Information

Put a date if patient withdrew study

____

Reason patient withdrew from study

- ◯ Non-compliance
- ◯ Did not wish to continue in study
- ◯ Could not tolerate the study intervention
- ◯ Hospitalization
- ◯ Other

Date of study completion

____

General Comments

Comments

____

2. Comments

MMR RCT - Comments/Notes

Date	Comments/Notes
______	______
______	______
______	______
______	______
______	______
______	______
______	______
______	______
______	______
______	______

Country of birth and Measles survey

Kia ora [first_names],

To help with the analysis of data within the MAXXED study, we would like to ask you to complete a short survey relating to your country of birth and any previous measles infection and/or immunisations.

This will enhance the quality of the data you have already contributed to during your study participation.

Warm regards,

MAXXED study team

Measles infection

Have you ever experienced a measles infection?	◯ yes - I have definitely had measles<br>◯ no - I have definitely NOT had measles<br>◯ I am unsure<br>(Any time prior to the recent blood screening test for your course entry)
Did you experience your measles infection while in Aotearoa, New Zealand?	◯ Yes<br>◯ No
Which country outside of Aotearoa, New Zealand were you living when you contracted measles?	______________________________

What year did this infection occur? ______
Was your measles infection confirmed by a medical diagnosis (eg. doctor assessment)? ______
Was your measles infection confirmed with a lab test? ______
Do you have any medical records relating to your measles diagnosis (eg. lab test results or other medical records)? ______

Measles Vaccinations

Please select the option describing your Measles vaccination history before entering your current course of study	◯ I received ONE dose of MMR during my childhood<br>◯ I received TWO doses of MMR during my childhood<br>◯ I DID NOT receive any doses of MMR during my childhood<br>◯ I am unsure
Were you in Aotearoa, New Zealand when you received your MMR doses in childhood?	◯ Yes<br>◯ No
In which country did you receive MMR vaccination (or other measles vaccination)?	______________________________
Do you recall whether your MMR vaccination record was provided to Student Health, or was a declaration of vaccination provided?	◯ I provided my vaccination records to Student Health<br>◯ I provided a declaration that I received MMR vaccination as I was unable to locate the written record<br>◯ I am not sure

Lab Screening Data - obselete

Please complete the survey below.

Thank you!

Base data from Lab Serology

Date of Test	________________
First name	________________
Surname	________________
Date of Birth	________________
Screening blood sample available?	◯ Yes<br>◯ No
SCL screening specimen ID	________________
Mumps antibody result	________________<br>(AU/ml)
Measles antibody result	________________<br>(AU/ml)
