# Supplementary material for: Immunogenicity and safety of measles-mumps-rubella vaccine delivered by the aerosol, intradermal and intramuscular routes in previously vaccinated young adults: a randomized controlled trial protocol
Source: PLoS One. 2025 Mar 21;20(3):e0318893. doi: 10.1371/journal.pone.0318893 (PMC11927902; doi:10.1371/journal.pone.0318893)
Supplement: S3 File — (PDF) [file pone.0318893.s003.pdf]

CONSENT FORM

Study:	**Measuring and boosting waning immunity to measles in young adults Vaccine Trial**
Locality:	University of Otago, Student Health Services, Dunedin
Lead Investigator:	Prof Peter McIntyre Ethics committee ref: 2022 FULL 13681

Please read the following statements before signing this form.

1. I have read the Participant Information Sheet and understand the aims and requirements of this research project.
2. I have had sufficient time to consider whether or not to participate in this study.
3. I have had the opportunity to talk with people of my choice about participating in the study.
4. I know that my participation in the project is entirely voluntary, and that I am free to withdraw from the project at any time without disadvantage.
5. I confirm that I meet the criteria for participation explained in the Participant Information Sheet.
6. I consent to the research staff collecting and processing my information, including information about my past vaccinations and screening blood results from Student Health Services and Southern Community Laboratories (SCL)
7. All my questions about the project have been answered to my satisfaction, and I understand that I am free to request further information at any stage.
8. If I decide to withdraw from the study, I agree that the information collected about me up to the point when I withdraw may continue to be processed.
9. I agree to be randomly assigned one of the three explained vaccine delivery methods.
10. I understand the risks of discomfort or harm explained in the Information Sheet.
11. I agree to donate my blood and oral fluid samples for analysis in this study, and understand when these will be required.
12. I understand that my blood samples will be sent to an overseas laboratory for analysis and will be kept there for up to 5 years.
13. I agree that Student Health Services will be informed about my participation in the study and of the relevant results from blood testing.

14. I know that when the project is completed all personal identifying information will be removed from the paper records and electronic files which represent the data from the project, and that these will be placed in secure storage and kept for at least ten years.
15. I understand that the results of the project may be published and be available in the University of Otago Library, but that any personal identifying information will remain confidential, and will not appear in any spoken or written report of the study.
16. I agree to an approved auditor appointed by the New Zealand Health and Disability Ethics Committees, or any relevant regulatory authority or their approved representative reviewing my relevant study and vaccination records for the sole purpose of checking the accuracy of the information recorded for the study.
17. I understand the arrangements for payment in compensation for time and inconvenience due to my participation in this study, and have had my questions about this addressed to my satisfaction.
18. I know who to contact if I have any questions about the study in general

19. I wish to receive a summary of the results from the study Yes☐ No☐

20. I am willing to be contacted for further research Yes☐ No☐

Declaration by participant:

I hereby consent to take part in this study

Participant's name:

Signature: Date:

Declaration by member of research team:

Researcher's name:

Signature: Date:
