# Supplementary material for: Immunogenicity and safety of measles-mumps-rubella vaccine delivered by the aerosol, intradermal and intramuscular routes in previously vaccinated young adults: a randomized controlled trial protocol
Source: PLoS One. 2025 Mar 21;20(3):e0318893. doi: 10.1371/journal.pone.0318893 (PMC11927902; doi:10.1371/journal.pone.0318893)
Supplement: S4 File — (PDF) [file pone.0318893.s004.pdf]

Vaccination devices used in the MAXXED (MeaslesvAXXroutEsofDelivery) trial

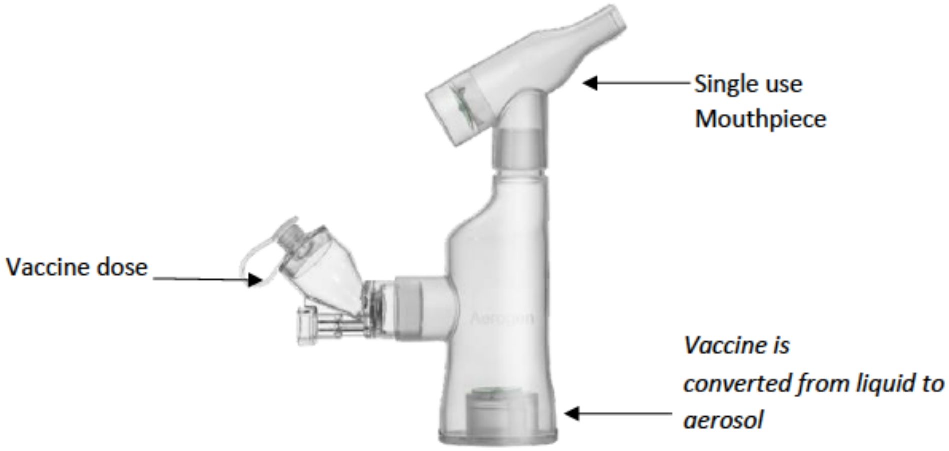

Figure 1. The Aerogen device designed for aerosol delivery of vaccine

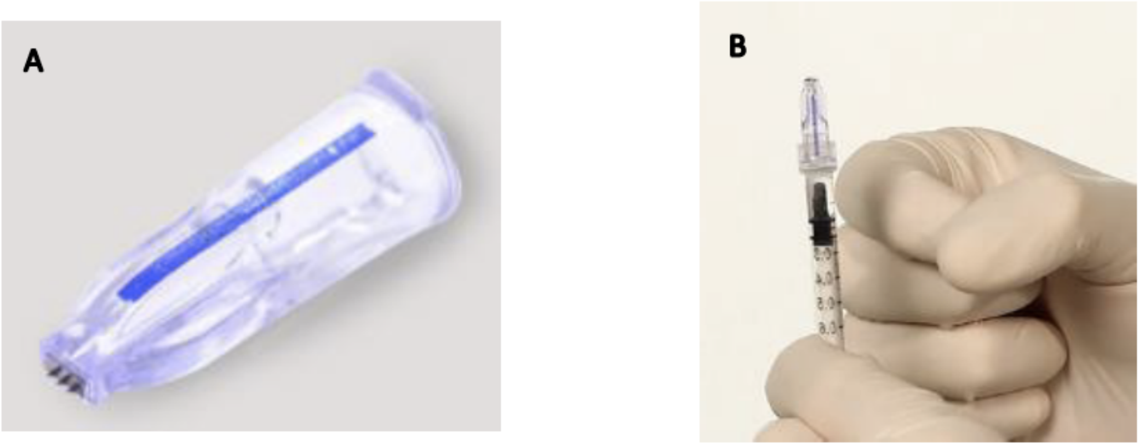

Figure 2. Components of the microneedle device used for intradermal vaccine delivery

A. Close-up view of microneedle tip. B. The assembled vaccine syringe and microneedle
