# Supplementary material for: Immunogenicity and safety of measles-mumps-rubella vaccine delivered by the aerosol, intradermal and intramuscular routes in previously vaccinated young adults: a randomized controlled trial protocol
Source: PLoS One. 2025 Mar 21;20(3):e0318893. doi: 10.1371/journal.pone.0318893 (PMC11927902; doi:10.1371/journal.pone.0318893)
Supplement: S1 Protocol — (PDF) [file pone.0318893.s005.pdf]

**Protocol Title:**	Measuring and boosting waning immunity to measles in young adults – Clinical Trial
**Protocol Number:**	MMR-100
**Study Type:**	
**Sponsor:**	Professor Peter McIntyre<br>Department of Women's and Children's Health<br>Dunedin School of Medicine<br>University of Otago
**Funding:**	Health Research Council
**Protocol Version:**	1.0
**Protocol Version Date:**	2nd December 2022

Table of Contents

1.0 PROTOCOL SUMMARY 5
2.0 BACKGROUND 8
3.0 RATIONALE 9
4.0 Purpose of the Study 11
5.0 OBJECTIVES AND ENDPOINTS 11
6.0 STUDY DESIGN 12
6.1 Overall Design 12
6.2 Eligibility 12
6.3 Initial contact by the study team 12
6.4 Pre enrolment screening 12
6.5 Schedule of Study procedures 13
6.6 Inclusion and exclusion criteria 13
6.7 Study Visit 1 (Day 0) - Procedures 14
6.7.1 Consent and enrolment 14
6.8 Subjects randomized to nebulized MMR delivery 14
6.9 Subjects randomized to intradermal MMR delivery (Nanopass) 15
6.10 Subjects randomized to intramuscular MMR 16
6.11 Post initial visit follow ups 17
6.11.1 Visit 2: (Day 3-4) 17
6.11.2 Visit 3: (Day 6-8) 17
6.11.3 Visit 4: (Day 13-15) 17
6.11.4 Visit 5: (Day 28-42) 18
6.11.5 Follow-up between day 28 and 12 months 18
6.11.6 Visit 6: (12 months) 18
6.12 Biological samples 18
6.13 Electronic Diary 19
6.14 Grading Scales 19
6.15 Local Reactions 19
6.15.1 Local Reaction Grading Scale 19
6.16 Systemic Events 20
6.16.1 Fever 21
6.16.2 Scale for Fever 21
6.17 Antipyretic Medication 21

6.18 Withdrawal Criteria ... 21
6.18.1 Participant Discontinuation Criteria ... 22
6.18.2 Withdrawal of Consent ... 22
6.18.3 Loss to Follow-up ... 22
7.0 SAFETY ASSESSMENTS ... 23
7.1 Adverse Events ... 23
7.2 Assessment of Unsolicited Adverse Events by the Investigator ... 23
7.3 Safety Monitoring ... 24
7.4 Serious Adverse Events ... 24
7.5 Serious Adverse Event Reporting − Procedures for Investigators ... 25
7.6 Pregnancy Reporting ... 25
7.7 Expedited Reporting ... 25
7.8 Safety Review Committee ... 26
8.0 Immunology assessments ... 27
8.1 Antibody responses ... 2727 27
8.2 Measles vaccine virus in oral fluid specimens ... 27
8.3 Return of results ... 27
9.0 DATA ANALYSIS AND STATISTICAL METHODS ... 28
9.1 Sample Size ... 28
9.2 Determination of Sample Size ... 28
9.3 General Statistical Methods ... 28
10.0 DATA MANAGEMENT AND RECORD KEEPING ... 29
10.1 Method of Participant Identifier Assignment ... 29
10.2 Reporting and Recording of Data ... 29
10.3 Medical Information Coding ... 29
10.4 Data Validation ... 29
11.0 INVESTIGATOR REQUIREMENTS AND QUALITY CONTROL ... 30
11.1 Regulatory Compliance ... 30
11.2 Ethics Committee (EC) Approval ... 30
11.3 Study Initiation ... 30
11.4 Informed Consent ... 30
11.5 Amendments and Deviations ... 31
11.5.1 Protocol Amendments ... 31

11.5.2 Emergency Deviations 31
11.5.3 Protocol Deviations 31
12.0 STUDY ADMINISTRATIVE INFORMATION 32
12.1 Study Monitoring Requirements 32
12.2 Case Report Forms 32
12.3 Data Management 32
12.4 Disclosure of Data 32
12.5 Retention of Records 32
12.6 Data Quality Assurance 33
12.7 Publications 33
12.8 SCOTT Assessment 33
13.0 REFERENCES 34
14.0 AppendiCES 36
14.1 Appendix 1. Abbreviations 36
14.2 Appendix 2. Safety Monitoring Committee 37

1.0 PROTOCOL SUMMARY

Protocol Title: Measuring and boosting waning immunity to measles in young adults

Rationale for research

Measles vaccine failure (VF) can be primary, where there is no antibody (Ab) response to measles, mumps and rubella (MMR) vaccine, or secondary (SVF), where protective levels of Ab wane over time such that clinical measles develop after exposure to measles virus. SVFs are being reported more often in countries with long-term high two dose MMR vaccine coverage and low measles circulation such as Aotearoa / NZ (12% of cases 20-29 years in 2019) – SVFs can transmit measles.

This study component addresses a key knowledge gap – can magnitude and duration of protection from MMR vaccine be improved by using non-standard routes of administration? An Italian study found that after a third dose of MMR (MMR3) using the conventional route (in Aotearoa intramuscular or IM), an Ab response above threshold is achieved in about 75% of young adults with waning Ab post MMR2, but their Ab levels are lower than those not requiring MMR3. Uncertainty about the strength and duration of protection achieved by MMR3 is the justification for exploring alternate modes of vaccine delivery, with the most promising being the aerosol and intradermal routes. **Aerosol delivery (AD*)*** of measles vaccine virus (MVV) improves ease of delivery and in school-aged children improved Ab responses, potentially even more using new-generation vibrating membrane devices (VMADs) but has not been assessed in seronegative young adults. A 2007 meta-analysis of MVV trials in older children randomised to SC or AD did not identify any safety signals, and superiority of AD is supported by data from non-human primates showing MVV by aerosol (but not injection or intra-nasally) protects against wild measles challenge. **Intradermal delivery (ID)** was found to significantly improve Ab responses to another live attenuated viral vaccine (zoster) over conventional. It has not been studied for MMR vaccine but is the theoretical basis for microneedle patches, an important innovation in measles vaccine delivery being evaluated worldwide.

Research design and methods

Each year, around 700 newly enrolling health professional students at the University of Otago who have a record of previous receipt of two doses of MMR are tested for Ab to measles (Me) and mumps (Mu), with ~30% requiring a dose of 3rd dose of MMR due to low Me or Mu (seronegative students).
Vaccine responses Consenting *seronegative students* are randomised to MMR administered AD, ID or conventional IM, while students electing not to participate in the randomized study but willing to make residual blood from their post-vaccine specimen available for study purposes will augment the data for immunogenicity in the IM group. The primary outcome is the proportion of subjects at 28 days post vaccination who achieve an Ab level >0.12 IU/ml for Me or >102 RIVM units for Mu, with a difference of more than 15% for comparison of AD with ID or IM considered clinically significant. Secondary outcomes are proportion achieving >=4-fold increase from baseline Ab and Geometric Mean Titres of Ab to Me and Mu at 28 days and 12 months post vaccine.

Research impact

This research offers benefits to Aotearoa/New Zealand, the Western Pacific region and globally through new knowledge about measles vaccine responses by delivery route, potentially applicable to other vaccines such as influenza and SARS-Cov-2. Had aerosol delivery been available, this could have been a valuable adjunct for mass immunisation, such as for the Auckland 2019 outbreak, and the MMR catch-up campaign underway across Aotearoa, both focused on Māori and Pacific youth because of disproportionately higher measles incidence and risk of complications. Innovative vaccine delivery (aerosol or id/microneedle patch) is potentially relevant for covid-19 vaccines, a challenge in mass delivery for Aotearoa and globally. Our team has a strong record of policy translation and paths to dissemination at national and international level.

Positive results from the trial will have significance for equity of healthcare delivery for our wider community and will work towards the commitment to Te Tiriti obligations. Future benefits for Māori

health care workforce are foreseeable in improving knowledge about, and strengthening protection against, measles – both for themselves and as a significant health need for whānau Māori generally, as shown in the 2019 outbreak. Simpler, non-needle-based delivery could reduce access and acceptance barriers, known to be significant contributors to under-immunisation. Intentional recruitment policies at the University of Otago aimed at increasing equity and representation (Mirror on Society) should ensure Māori students are well represented in the eligible cohort aiding explanatory power of the study.

Overall design

Assessing immunogenicity and acceptability of administration of MMR vaccine by nebulizer or intradermal device to subjects seronegative despite past receipt of two doses of MMR vaccine.

Brief summary

The purpose of this study is assessment of immunogenicity and tolerability of administration of MMR vaccine by the aerosol and intradermal (deltoid) routes compared with standard intramuscular. Based on previous years, we expect between 200 to 250 students commencing health professional courses at the University of Otago in 2023 to have antibody levels below threshold on either or both of the Diasorin assay (16.5 arbitrary units (AU) for measles or mumps (10.9 AU) and be required to receive a third dose of MMR according to university policy. These students will be offered the opportunity to participate in a trial to compare responses to MMR vaccine by delivery route.

Trial participants will be randomized to receive MMR given by a) standard intramuscular, b) nebulized aerosol or c) intradermally in the deltoid region. Students who do not wish to participate in the randomized study of one of these two novel routes can receive MMR by standard intramuscular administration and be enrolled if willing to make their post MMR blood sample available for the study. Primary outcomes are proportion reaching the antibody threshold for protection and proportion achieving a four-fold or greater increase from baseline antibody level at 28 days post vaccination for each route of administration. Secondary outcomes are GMT of measles and mumps antibody at 28 days, and after 12 months.

Study procedures

- Eligibility (section 6.2), inclusion and exclusion criteria assessed and met (section 6.6)
- Randomization to either Intramuscular, Aerosol or Intradermal MMR vaccine delivery
- Administering 0.3 mL Priorix MMR vaccine via either a) intramuscular, inhaled aerosol or intradermal delivery
- Electronic collection of safety reports via mobile phone at Days 4, 7 and 14
- Collection of oral fluid: Days 4, 7 and 14
- Blood sample: Day 28, with sampling up to day 42 eligible for study
- Contact maintained by study staff with participants over the next 12 months
- Blood sample at 12 months post first study MMR vaccination (MMR3)

Study population and number of participants

Based on figures in 2022 for Otago students, about 250 will require MMR. If 40 to 60% consent to participate in the randomised study, this will be 100 to 150 in total or 30 to 50 per arm (aerosol, intradermal, intramuscular) in year 1. Based on participant recruitment and serological outcomes in year 1, recruitment targets will be reviewed for year 2, with a target of 100 per arm across two years. Of the remaining 100 to 150 students required to have MMR, we anticipate that at 50 to 70% or 50 to 105 per year will agree to allow the study to have access to their pre vaccine screening blood sample and their post vaccine blood sample, or 100 to 210 across 2 years. If a 15% difference (77% vs 92%) in vaccine response is considered clinically important, we require 87 participants per group to detect a difference

at least this large with alpha=0.05 and power=80%. Allowing for 10% drop-out, this becomes 97 participants per group, which is within our anticipated recruitment target.

We will evaluate recruitment and seroconversion in year 1 and take measures to increase recruitment if necessary for year two.

Safety Review Committee

Has been established to review participant responses weekly during active recruitment, with data available from mobile phone-based prompts. Members: Professor Michael Tatley; Dr Jennifer Lee, New Zealand Pharmacovigilance Centre, University of Otago.

2.0 BACKGROUND

Waning immunity to measles is a potentially significant emerging problem in countries which have achieved elimination or near elimination of local transmission for long periods. After recovery from wild measles virus (WMV) infection, protection is lifelong, albeit with high short-term morbidity. (1) In contrast, antibody (Ab) levels induced by measles vaccine viruses (MVV) may wane to below protective levels if boosting from exposure to measles infection is uncommon, (2, 3) as in the last 20 years in Aotearoa. Measles vaccine failure (VF) - the occurrence of measles in vaccinated individuals - can be primary (PVF), where there was no Ab response post receipt of combined measles, mumps and rubella (MMR) vaccine, or secondary (SVF) where a primary Ab response occurs post MMR but wanes over time to non-protective levels, allowing clinical measles to develop after exposure. PVF, which occurs in up to 10% of MMR dose1 recipients, is the rationale for routinely giving a second dose (MMR2). (2,3) SVFs are being reported more often in countries with long-term high MMR2 and low measles circulation. (1,3,4) In the 2019 epidemic in Aotearoa, cases were concentrated by geography (86% from Northern, Waitemata, Auckland and Counties Manukau DHBs) and ethnicity (24% Māori and 41% Pasifika), primarily related to under-vaccination.(5) Importantly, of the total of 2174 cases, 32% were aged 20 to 29 years, among whom 12% had two doses of MMR vaccine ie SVFs.(5) Although the risk of infection and transmission of measles is significantly lower in persons with 2 prior MMR doses, both are well documented,(1,3) and a particular issue for Health Care Workers (HCWs) with a higher probability of exposure.(6) In summary, evidence is accumulating of increasing measles susceptibility in fully vaccinated people in Aotearoa. Although high two dose measles vaccine coverage is key, while measles outbreaks continue at global level (including European countries), this poses a threat to elimination in Aotearoa and similar settings. (3) Hence, the outcomes of this research - looking to see how strong protection from vaccination is over time and if it can be improved - of key importance to communities who were disproportionally affected and who have the most potential benefit and impact ie: whānau Māori.

What immune correlates predict protection against measles?

A 2020 systematic review found 5 methodologically adequate studies, but estimates of protective levels of antibody were problematic because of assay variability and differing criteria for measles infection (clinical vs 4 fold antibody rise post exposure).(7) Although some authors have argued for an absolute threshold for antibody protection, evidence favours progressive susceptibility on a sliding scale.(7) The seminal study, conducted in the US pre-elimination, examined immunised students in a residential college who had blood samples available prior to a measles outbreak. All 8 clinically typical cases had pre-exposure titres <1:120 by the plaque reduction neutralisation test (PRNT) and undetectable Ab by standard ELISA. Among those with titres >1:120 but <1:1052, 26/37 (70%) had at least one symptom vs 0/7 >1:1052, and 7/11 (64%) with intermediate titres (1:216 to 1:874) had a 4-fold antibody rise. (8) A more recent study in an elimination setting (Netherlands) reported that 2/5 immunised HCWs developed symptomatic infection despite PRNT titres >1:120. (9) Transmission of measles to others, including fully immunised people, from symptomatic cases with documented MMR2 has been documented, emphasising the potential clinical and public health importance of waning immunity. (1,10)

Is improved vaccine protection possible?

A third dose of MMR (MMR3), given by the conventional subcutaneous (SC) route, is the logical first step to improving vaccine protection against measles (Me), mumps (Mu) or rubella (Ru), but studies are limited. In a US cohort studied more than 10 years after receipt of the second of two MMR doses, only 24/662 (4%) had low measles IgG ; 16/24 (75%) responded post MMR3 whereas those with higher antibody did not.(9) Among health care students in Italy, of 212 with

CONFIDENTIAL

low IgG despite two MMR doses (15% of total students), 157 (74%; 95% CI, 68–80%]) had an IgG response post MMR3, but geometric mean titre (GMT) was significantly lower (46U/ml) than those who were seropositive post MMR2 (77U/ml).(10) Among 36 students who remained seronegative after MMR3, only 13 (36% [95% CI 21–54%]) seroconverted. Currently, the best evidence for the level of antibody for protection against clinical measles is ≥120 IU of neutralising antibody, based on a small study in the pre-elimination era in the US,(11) but it is unknown how this applies to current commercial assays (6) or in the post-elimination setting.(12) Exploratory research such as this provides the opportunity to have confidence in these new methods of delivery if they are found to be safe and effective vaccines, for a preventative disease with significant morbidity and mortality in communities.

Interpretation of antibody assays for measles, mumps and rubella

The PRNT assay is regarded as a 'gold standard' laboratory method for immunogenicity studies of measles vaccine because it is a functional antibody assay, measuring neutralising antibodies. PRNT has the disadvantages of being technically demanding, labour intensive and time-consuming and, as a variable bio-assay, is difficult to standardise between different laboratories.

Enzyme-linked immunosorbent assays (ELISAs), on the other hand, are rapid, simple to perform, relatively inexpensive and easier to standardise. A recent US study examined the sensitivity and specificity of ELISA assays in a common set of sera against a PRNT assay calibrated using WHO standards. Discrepant results occurred in 15-20% of samples, most marked at the lowest titres typically found among previously vaccinated subjects. (27) The multiplex bead-based immunoassay (MIA) developed by RIVM (Netherlands) overcomes this problem by calibration against the WHO 3rd international standard serum for measles expressed in mIU/ml [28]. A similar approach was undertaken for measurement of antibody levels against mumps and rubella. (14)

3.0 RATIONALE

Alternate delivery routes could improve antibody response and longevity

1. ***Aerosol delivery (AD)*** **of MVV** (monovalent or with rubella) has been used routinely to deliver a second dose around school entry to around 4 million children in Mexico and studied in multiple RCTs.(17) In one RCT in Mexico (N=562), AD yielded significantly higher seroconversion (99% vs 82%) than SC with significantly fewer adverse reactions (fever 1.6% vs 6.5%, cough 0.4% vs 17.2%) and in another (N=1624) >50% had a 4 fold rise in titre with AD vs 4-23% with SC. In a study in South Africa led by one of our investigators (FC), children 5-14 years were randomised to receive the Edmonston-Zagreb (EZ) strain of MV by AD or SC and Schwartz MV (the strain in the MMR currently used in Aotearoa/NZ).

Among children defined as seronegative, only 8% of 160 in EZAD group remained seronegative one year later vs 19% of 128 for EZSC (P=0.004) and 27% of 128 for SWSC (P<0.001). (18) For vaccines also including mumps (MMR) and in adults, data are limited to 3 studies in Mexico with most subjects seropositive, only one of which used a vaccine with virus strains similar to those used in Aotearoa (MMRII). (19) MMRII was given to 27 adults 21-38 years, of whom most were seropositive (measles 96%; mumps 70%). For measles, a two-fold rise in titre was seen for 12/18 (67%) in the lowest bands of Ab titre vs 0/9 in the highest band and for mumps in 9/18 (50%) vs 2/9 (22%). Overall, GMTs increased significantly from baseline. (19)

The logistics and safety of aerosol delivery of MMR vaccine is key to wider applicability.

The Mexican and South African studies used a bulky home-made device. (17) A WHO-sponsored RCT in infants in India in 2009 was preceded by a process of selection of modern nebulisers for

aerosol vaccine delivery. (20) These studies found that the potency of vaccine virus was preserved after nebulisation by modern jet or vibrating mesh nebulisers and resulted in the selection of the vibrating mesh nebuliser manufactured by Aerogen, being used in this study, for use in the WHO RCT. (23) The Aerogen nebuliser has been shown to release only 0.2% of aerosolised drug into the environment, with no evidence of infection by medical nebulisers during the SARS epidemic in 2009. (24) In a study in Mexico including adults, children and nurses administering aerosolised MMR vaccine, there was no evidence of transmission of vaccine virus to contacts or administrators. (22) A 2007 meta-analysis of MV trials in children randomised to SC or AD aerosol did not identify any safety signals (21) and similarly later studies in adults. (19,22)

2. Intradermal delivery (ID) has been shown to be effective among non-responders to inactivated vaccines given intramuscularly, such as Hepatitis B. A recent study found significantly higher immune responses to live attenuated zoster vaccine (Zostavax, currently used in Aotearoa for adults over 65 years) with ID delivery using a specifically designed device (Nanopass) vs standard SC. (25) ID delivery has not been studied for other live attenuated vaccines, including MMR vaccine, but is the theoretical basis for development of microneedle patches, a new technology for delivery of measles and rubella vaccines. (26)

ID delivery is typically associated with more evident local induration and erythema at the injection site due to the deposition of fluid and vaccine more superficially than either subcutaneous or intramuscular administration. In the study of ID delivery of Zostavax in older adults, there was significantly greater erythema among vaccinees, but injection site pain was reported by the similar proportions of recipients of full dose ID (24%) or subcutaneous (29%) vaccine.

4.0 PURPOSE OF THE STUDY

The purpose of the study is to compare the immunogenicity and reactogenicity of MMR vaccine delivered by aerosol or intradermally (deltoid injection site) with conventional IM administration

The study will assess immunogenicity and acceptability among consenting seronegative participants of vaccination using the aerosol and intradermal routes by measurement of antibody using a microimmune assay (MIA) at RIVM (Netherlands Public Health Institute), with confirmatory plaque reduction neutralization assay (PRNT) assays in a subset. The primary immunogenicity outcomes are seroresponse to above the threshold for protection and fourfold increase from baseline at 28 days post MMR. The primary reactogenicity outcomes are local or systemic symptoms reported by recipients using electronic prompts at 48 hours and 7 days. The secondary outcomes of GMT at 28 days and 12 months.

5.0 OBJECTIVES AND ENDPOINTS

Table 1

Objectives	Endpoints
Primary	
• Immunogenicity and reactogenicity of MMR vaccine delivered by aerosol device in seronegative students	• Proportion of participants who exceed the cut-off for seropositivity by MIA and PRNT assays at RIVM<br>• Proportion of participants who have a two and fourfold rise in MIA and PRNT antibody<br>• Proportion of participants who report no adverse events in at 1-, 3-, 7- and 14-days post MMR
• Immunogenicity and reactogenicity of MMR vaccine by intradermal device in seronegative students	• Proportion of participants who exceed the cut-off for seropositivity by MIA and PRNT assays at RIVM<br>• Proportion of participants who have a two and fourfold rise in MIA and PRNT antibody<br>• Proportion of participants who report no adverse events at 1-, 3-, 7- and 14-days post MMR<br>•
Secondary	
• Short- and longer-term immunogenicity of MMR vaccine delivered by aerosol or intradermally in seronegative students<br>• Detection of measles vaccine virus (MVV) in oral fluid by PCR at 3-, 7- and 14-days post vaccination	• Geometric Mean Titre (GMT) at 28 days and 12 months post MMR<br>• % in whom MVV is detected by PCR of oral fluid at each time point

CONFIDENTIAL

6.0
6.0 STUDY DESIGN

6.1 **Overall Design**

Assessing immunogenicity and reactogenicity of administration of MMR vaccine by nebulizer or intradermal device (at the deltoid) to subjects seronegative despite past receipt of two doses of MMR vaccine

6.2 Eligibility

Students at the University of Otago, Dunedin, who have been accepted into professional health science courses to which the Screening and Immunisation Policy applies (Medicine, Medical Laboratory Science, Physiotherapy, Pharmacy and Dentistry) are eligible for this study if course-required screening serology returns results to indicate they are below the cutoff for positive and equivocal measles and mumps results (<= 16.5 AU for measles and <= 10.9 AU for mumps, using the Diasorin assay). All such students will be required to receive MMR vaccine by University policy.
In a pilot study undertaken in 2021, Southern Community Laboratories (SCL) stored these screening blood specimens tests for up to 3 months. If during this time, informed consent to retaining these specimens was obtained from students they could be used as the baseline sample [Health and Disability Southern Ethics Committee (HDEC) January 2021 (21/STH/80).] A similar arrangement will be sought for this study.

6.3 **Initial contact by the study team**

University of Otago Student Health Services are responsible for receiving results of screening antibody results from SCL and indicating to students if they are required to receive MMR vaccine. In the same way as the earlier pilot (HDEC 21/STH/80), it is proposed that Student Health would indicate, when contacting students required to receive MMR based on their screening blood test, that this study is commencing in 2023 and is funded by the Health Research Council. In the email from Student Health, a brief summary of the rationale for the study, its benefits, requirements and options for participation will be provided. It will be indicated that participants in the randomised study (4 study visits over 4 weeks and a follow-up at 12 months) are compensated with a payment of $25 for each visit and have the cost of their post MMR blood tests (required under Otago policy) paid (approximately $33). Students interested in further information are asked to click on a link to indicate yes/no if they wish to receive additional information about the study. Students who click yes are taken to links providing additional information about the study and asked to provide contact details for e mail or text contact from study staff. Students who have not responded either yes or no within 14 days are sent one reminder email on our behalf from Student Health.

6.4 **Pre enrolment screening**

- Eligible students who indicate they wish to view more detailed study materials are sent the information sheet, options for participation in the study and consent form to view, with a request to nominate a time to discuss potential enrolment by zoom, or in-person if desired.
- Students who are willing to participate are sent a link to video demonstration of study procedures and to make a time for pre-enrolment zoom interview.

- During the pre-enrolment zoom interview:
    - Additional explanation is given of study procedures:

- Willing to be allocated to aerosol, intradermal or intramuscular administration, make demographic and immunisation data provided to student health available to the study, attend all 5 study visits - days 3-5, 6-8, 13-15, 28-42 and at 12 months - and undergo related testing.
- Participants are taken through the patient information sheet and consent form and have any questions addressed. If they agree in principle to participation in one of the study arms, they are given an appointment for their first study visit and asked to complete the consent form online.

- Final questions, confirmation of participation, and signing of the consent form occur at the first study appointment.

6.5 Schedule of Study procedures

Table 2

Day	0	4<br>3-5[b]	7[b,c]<br>[6-8]	14<br>[13-15]	30<br>[28 to 42]	12 months
Visit number	1	2	3	4	5	6
Visit description	Screening/ enrollment/ vaccination		7-day follow-up			
**Procedures**						
Written informed consent	X					
Enrollment	X					
Solicited reactogenicity collection		X	X		X	
Adverse Event collection		X[b]	X	X		
Vaccine acceptability assessments			X[b]		X[b]	
Serum collection	X[a]		X[c]	X[c]	X[d]	X[c]
Oral fluid collection by Oracol oral swab		X	X	X		

Footnotes
a: only if pre-vaccine screening specimen not available – via fingerprick
b: Electronic prompts days 1-3 and day 7, 14. Safety Committee review of day 7 and day 28 outcomes
c. via fingerprick, or venepuncture blood draw if more appropriate
d. via blood draw – also used for routine laboratory testing of measles antibody

6.6 Inclusion and exclusion criteria

- *Eligibility and inclusion criteria:*
  - Antibody below Diasorin threshold for either or both of measles/mumps antibody; required to have a dose of MMR vaccine by Otago Screening and Immunisation Policy
  - Capable and willing to give written informed consent
  - Residing in Dunedin
  - Able and willing to participate for the duration of the study visits and follow-up
  - Willing to provide verifiable identification at study entry and follow-up visits
  - Daily access to an internet-connected device (smart phone, tablet, laptop or PC) and willing to complete an electronic diary post vaccination.

CONFIDENTIAL

Exclusion criteria:

- Acute illness within 5 days prior to study vaccination
- Contraindications to MMR as specified in the Aotearoa / NZ Immunisation Handbook. These include:
    - Proven anaphylaxis to the vaccine or vaccine component (eg, neomycin or gelatin)
    - Significant immunocompromise: impaired cell-mediated immunity, including untreated malignancy, type 1 interferon receptor (IFNAR) signalling pathway defects, immunosuppressive drug therapy, including high-dose steroids, receiving high-dose radiotherapy, HIV infection with severely impaired T cell immunity
    - Another live vaccine, including BCG, within the previous 4 weeks
    - Pregnant women – pregnancy should be avoided for four weeks after immunization
    - Participants pregnant during study participation may complete follow-up.
    - Intravenous immunoglobulin or blood transfusion during the preceding 11 months

6.7 Study Visit 1 (Day 0) - Procedures

6.7.1 Consent and enrolment

Obtain a record of informed consent prior and check that documentation below is available:

- All inclusion criteria and none of the exclusion criteria are met
- Medical history of clinical significance, with particular reference to asthma or any respiratory conditions
- Smoking and vaping history.
- Medications taken currently and in the 28 days prior to enrolment.
- Vaccinations within 28 days prior to enrolment
- Once above confirmed as complete, randomize and assign a unique participant number.
- Obtain vital signs (weight, height, body temperature, pulse rate)
- Record vaccinations to be given in addition to MMR (eg Hepatitis B, varicella)
- Confirm that a stored serum taken in the previous 3 months is available for study use.
    - If not available, collect a blood sample for study use by fingerprick
- Demonstrate what is involved in collecting oral fluid sample via directing to information on mouth swab technique
- According to allocation to aerosol or intradermal provide information about vaccine administration and seek any questions.

6.8 Subjects randomized to nebulized MMR delivery

- Prepare Priorix (MMR) for aerosol delivery by adding 0.3 ml of diluent included in the syringe to freeze-dried vaccine in ampoule and mixing thoroughly
- Select a new set of administration equipment (container for reconstituted vaccine and connector to electronic controller + nozzle and chamber for subject to inhale and exhale)
- Withdraw reconstituted vaccine from ampoule (0.3 ml) and inject into the container for Aerogen Solo

- After the subject has inserted the nozzle into their mouth and started breathing, use the blue button on the controller to commence nebulisation. Inhale and exhale slowly, repeating as often as necessary until no residual liquid remains in the Aerogen Solo cup. This is expected to take 30 to 60 seconds.
- After immunization, press the power on / off button and the indicator light goes out.

Notes for attention

- Issue a thermometer for recording daily temperatures and provide instructions on use.
- Explain the e-diary technologies available for this study and assist the participant in downloading the study application onto their own device (mobile phone, laptop, ipad etc). Provide instructions on e-diary completion and ask the participant to complete the reactogenicity e-diary from Day 1 to Day 7, with Day 1 being the day of vaccination.
- Ask the participant to contact the site staff or investigator immediately if he or she experiences any of the following from Day 1 to Day 7 after vaccination (where Day 1 is the day of vaccination) to determine if an unscheduled reactogenicity visit is required:
    - Fever ≥ 39.0°C
    - Severe pain at the injection site.
    - Any systemic event which meets study criteria for a sever event.
- Ask the participant to contact the site staff or investigator if a medically attended event (eg, doctor’s visit, emergency room visit) or hospitalization occurs.
- Schedule an appointment for the participant to return for the next study visit (day 3-5)
- The investigator or an authorized designee completes the CRFs.
- The investigator or qualified designee reviews the reactogenicity e-diary data online following vaccination to evaluate participant compliance and as part of the ongoing safety review.

6.9 Subjects randomized to intradermal MMR delivery (Nanopass)

- Prepare Priorix (MMR) for intradermal delivery by adding 0.3 ml of diluent included in the syringe to freeze-dried vaccine in ampoule and mixing thoroughly
- Withdraw reconstituted vaccine from ampoule into a 1 ml syringe and attach the Nanopass device
- Hold syringe between thumb and forefinger and align device using the blue line indicator
- Insert needle into skin at 45° to the skin in the deltoid region
- Use gentle pressure to inject syringe contents until a visible bleb is raised.
- Withdraw syringe

Notes for attention

- Issue a ruler for measurement of local reaction size and redness
- Issue a thermometer for recording daily temperatures and provide instructions on use.
- Explain the e-diary technologies available for this study and assist the participant in downloading the study application onto the participant’s own device (mobile phone, laptop, ipad etc). Provide instructions on e-diary completion and ask the participant to complete the reactogenicity e-diary from Day 0 to Day 7, with Day 0 being the day of vaccination.

- Ask the participant to contact the site staff or investigator immediately if he or she experiences any of the following from Day 0 to Day 7 after vaccination (where Day 0 is the day of vaccination) to determine if an unscheduled reactogenicity visit is required:
  - Fever ≥ 39.0°C
  - Severe pain at the injection site.
  - Any systemic event which meets study citeria for a severe event.
- Ask the participant to contact the site staff or investigator if a medically attended event (eg, doctor's visit, emergency room visit) or hospitalization occurs.
- Schedule an appointment for the participant to return for the next study visit and advise the participant of the telephone contact Day 8 to review e-diary and assess adverse events.
- The investigator or authorized designee completes the CRFs.
- The investigator or qualified designee reviews the reactogenicity e-diary data online following vaccination to evaluate participant compliance and as part of the ongoing safety review.

6.10 Subjects randomized to intramuscular MMR

- Prepare Priorix (MMR) for intramuscular delivery but add 0.3 ml (not 0.5 ml) of diluent included in the syringe to freeze-dried vaccine in ampoule and mix thoroughly
- Withdraw reconstituted vaccine from ampoule into a 1 ml syringe and administer using routine 23G needle by intramuscular injection
- Withdraw syringe

Notes for attention

- Issue a ruler for measurement of local reaction size and redness
- Issue a thermometer for recording daily temperatures and provide instructions on use.
- Explain the e-diary technologies available for this study and assist the participant in downloading the study application onto the participant's own device (mobile phone, laptop, ipad etc). Provide instructions on e-diary completion and ask the participant to complete the reactogenicity e-diary from Day 0 to Day 7, with Day 0 being the day of vaccination.
- Ask the participant to contact the site staff or investigator immediately if he or she experiences any of the following from Day 0 to Day 7 after vaccination (where Day 0 is the day of vaccination) to determine if an unscheduled reactogenicity visit is required:
  - Fever ≥ 39.0°C
  - Severe pain at the injection site.
  - Any systemic event which meets study criteria for a severe event.
- Ask the participant to contact the site staff or investigator if a medically attended event (eg, doctor's visit, emergency room visit) or hospitalization occurs.
- Schedule an appointment for the participant to return for the next study visit and advise the participant of the telephone contact Day 8 to review e-diary and assess adverse events.
- The investigator or authorized designee completes the CRFs.
- The investigator or qualified designee reviews the reactogenicity e-diary data online following vaccination to evaluate participant compliance and as part of the ongoing safety review.

6.11 Post initial visit follow ups

Participants will receive digital reminders to enter diary information at 12 and 24 hours and daily to day 7 and to return to Student Health on day 3-4, 6-8 and 12-14. If digital responses not received, participants will be followed up by study staff via text to their mobile phone number.

The clinical research nurse will inform Student Health Services of the study participants who receive MMR vaccine, the lot# of the vaccine, and the method of delivery.

6.11.1 Visit 2: (Day 3-4)

- Review e diary and check for any unreported adverse events
- Collect an oral fluid sample using the Oracol pro device
- Schedule an appointment for the next study visit (day 6-8)
- The investigator or an authorized designee completes the CRFs
- The investigator or appropriately qualified designee reviews the reactogenicity e-diary data

6.11.2 Visit 3: (Day 6-8)

- Review e diary and check for any unreported adverse events
- Collect an oral fluid sample using the Oracol pro device
- Collect a blood sample (100 microlitre minimum) by finger prick
- Label oral fluid and blood samples with participant details
- Schedule an appointment for the next study visit (day 13-15)
- The investigator or an authorized designee completes the CRFs
- The investigator or appropriately qualified designee reviews the reactogenicity e-diary data

6.11.3 Visit 4: (Day 13-15)

- Review e diary and check for any unreported adverse events
- Collect an oral fluid sample using the Oracol pro device
- Collect a blood sample (100 microlitre minimum) by finger prick
- Label oral fluid and blood samples with participant details
- Advise procedure for next study visit (day 28-42) ascertaining if Hepatitis B serology required
- The investigator or an authorized designee completes the CRFs
- The investigator or appropriately qualified designee reviews the reactogenicity e-diary data

6.11.4 Visit 5: (Day 28-42)

- Ascertain that blood sample has been collected at SCL laboratory site
- Check that all study data is complete
- Arrange for reimbursement of laboratory and other costs
- Label oral fluid and blood samples with participant details
- Schedule an appointment for study visit 12 months later
- The investigator or an authorized designee completes the CRFs

6.11.5 Follow-up between day 28 and 12 months

- Ensure participant has been notified about local laboratory measles antibody results
- Ensure participant has been notified about Netherlands laboratory measles antibody results
- Check in at 3 monthly intervals to remind about 12 month follow up

6.11.6 Visit 6: (12 months)

- Contact participant to finalise arrangements for final blood collection
- Arrange for reimbursement for final visit
- Check participant contact details for test results

6.12 **Biological samples**

Blood and oral fluid samples will be used only for scientific research. Each sample will be labeled with a code so that the laboratory personnel testing the samples will not know the participant's identity. Testing of the day 28 serum specimen will be performed by Southern Community Laboratories Dunedin using the Diasorin assay, to provide rapid feedback to participants and for the Student Health Service to follow up as required by the University of Otago Screening and Immunisation Policy.

All samples (blood and oral fluid) for each year's cohort of subjects will be shipped to RIVM (Netherlands Public Health Laboratory) Bilthoven, Netherlands once available. Samples that remain after performing assays outlined in the protocol, if participants agree to future use of their specimens at the time of informed consent, may be stored at RIVM for up to 5 years after the end of the study and then destroyed. The participant may request that their samples, if still identifiable, be destroyed at any time; however, any data already collected from those samples will still be used for this research. Any testing of samples not described in the future use consent form will require additional approval from an ethics committee.

6.13 Electronic Diary

Participants will be requested to complete a reactogenicity e-diary through a web-based application accessible on the participant's own personal device (see Section 7.11.1). All participants will be asked to monitor and record local reactions, systemic events, and antipyretic medication usage for 7 days following vaccine administration. The reactogenicity e-diary allows recording of these assessments only within a fixed time window, thus providing the accurate representation of the participant's experience at that time. Data on local reactions and systemic events reported in the reactogenicity e-diary will be available for review by investigators at all times via REDCap software. These data do not need to be reported by the investigator in the CRF as AEs. Investigators (or designee) will be required to review the reactogenicity e-diary data online as part of the ongoing safety review. The investigator or designee must obtain stop dates from the participant for any ongoing local reactions, systemic events, or use of antipyretic medication on the last day that the reactogenicity e-diary was completed. The stop dates should be documented in the source documents and the information entered in the CRF.

6.14 Grading Scales

The grading scales used in this study to assess local reactions and systemic events as described below are derived from the FDA Center for Biologics Evaluation and Research (CBER) guidelines on toxicity grading scales for healthy adult volunteers enrolled in preventive vaccine clinical trials.

6.15 Local Reactions

During the reactogenicity e-diary reporting period, participants will be asked to assess redness, swelling, and pain at the injection site and to record the symptoms in the reactogenicity e-diary. If a local reaction persists beyond the end of the reactogenicity e-diary period following vaccination, the participant will be requested to report that information. The investigator will enter this additional information in the CRF. Redness and swelling will be measured and recorded in centimeters and categorized as absent, mild, moderate, or severe based on the grading scale in Table 1. Pain at the injection site will be assessed by the participant as absent, mild, moderate, or severe according to the grading scale in Table 1.

If a Grade 3 local reaction is reported in the reactogenicity e-diary, a telephone contact should occur to ascertain further details and determine whether a site visit is clinically indicated. Only an investigator or medically qualified person is able to classify a participant's local reaction as Grade 4.

6.15.1 Local Reaction Grading Scale

Table 3

	**Mild (Grade 1)**	**Moderate (Grade 2)**	**Severe (Grade 3)**	**Potentially Life Threatening (Grade 4)**
**Pain at the injection site**	Does not interfere with activity	Interferes with activity	Prevents daily activity	Emergency room visit or hospitalization for severe pain
**Redness**	>2.0 cm to 5.0 cm	>5.0 cm to 10.0 cm	>10 cm	Necrosis or exfoliative dermatitis
**Swelling**	>2.0 cm to 5.0 cm	>5.0 cm to 10.0 cm	>10 cm	Necrosis

CONFIDENTIAL

6.16 **Systemic Events**

During the reactogenicity e-diary reporting period, participants will be asked to assess vomiting, diarrhea, cough, headache, fatigue, chills, new or worsened muscle pain, new or worsened rash or joint pain and to record the symptoms in the reactogenicity e-diary. The symptoms will be assessed by the participant as absent, mild, moderate, or severe according to the grading scale in Table 2.

If a Grade 3 systemic event is reported in the reactogenicity e-diary, a telephone contact should occur to ascertain further details and determine whether a site visit is clinically indicated. Only an investigator or medically qualified person is able to classify a participant's systemic event as Grade 4. If a participant experiences a confirmed Grade 4 systemic event, the investigator must immediately notify CARM.

Table 4. Systemic Event Grading Scale

	**Mild (Grade 1)**	**Moderate (Grade 2)**	**Severe (Grade 3)**	**Potentially Life Threatening (Grade 4)**
**Vomiting**	1-2 times in 24 hours	>2 times in 24 hours	Requires IV hydration	Emergency room visit or hospitalization for hypotensive shock
**Diarrhea**	2 to 3 loose stools in 24 hours	4 to 5 loose stools in 24 hours	6 or more loose stools in 24 hours	Emergency room visit or hospitalization for severe diarrhea
**Headache**	Does not interfere with activity	Some interference with activity	Prevents daily routine activity	Emergency room visit or hospitalization for severe headache
**Fatigue/ tiredness**	Does not interfere with activity	Some interference with activity	Prevents daily routine activity	Emergency room visit or hospitalization for severe fatigue
**Chills**	Does not interfere with activity	Some interference with activity	Prevents daily routine activity	Emergency room visit or hospitalization for severe chills
**New or worsened rash**	Does not interfere with activity	Some interference with activity	Prevents daily routine activity	Emergency room visit or hospitalization for new or worsened rash
**New or worsened muscle or joint pain**	Does not interfere with activity	Some interference with activity	Prevents daily routine activity	Emergency room visit or hospitalization for severe new or worsened muscle pain

CONFIDENTIAL

6.16.1 Fever

In order to record information on fever, a thermometer will be given to participants with instructions on how to measure oral temperature at home. Temperature will be collected in the reactogenicity e-diary in the evening daily during the reactogenicity e-diary reporting period. It will also be collected at any time during the reactogenicity e-diary data collection periods when fever is suspected. Fever is defined as an oral temperature of ≥ 38.0°C. The highest temperature for each day will be recorded in the reactogenicity e-diary. Temperature will be measured and recorded to 1 decimal place and then categorized during analysis according to the scale shown in Table 3.

If a fever of ≥ 39.0°C is reported in the reactogenicity e-diary, a telephone contact should occur to ascertain further details and determine whether a site visit is clinically indicated. Only an investigator or medically qualified person is able to confirm a participant's fever as >40.0°C. If a participant experiences a confirmed fever >40.0°C (>104.0°F), the investigator must immediately notify CARM and the participant's nominated medical care provider.

6.16.2 Scale for Fever

Table 5

Grade 1	≥38.0-38.4°C
Grade 2	>38.4-38.9°C
Grade 3	>38.9-40.0°C
Grace 4	>40.0°C

6.17 **Antipyretic Medication**

The use of antipyretic medication to treat symptoms associated with study intervention administration will be recorded in the reactogenicity e-diary daily during the reporting period (Day 1 to Day 7).

6.18 **Withdrawal Criteria**

In rare instances, it may be necessary for a participant to permanently discontinue study intervention (definitive discontinuation). Reasons for definitive discontinuation of study intervention may include the following: AEs; participant request; investigator request; pregnancy; protocol deviation (including no longer meeting all the inclusion criteria or meeting 1 or more exclusion criteria). In general, unless the investigator considers it unsafe to administer the second dose, or the participant does not wish to receive it, it is preferred that the second dose be administered.

6.18.1 Participant Discontinuation Criteria

A participant may withdraw from the study at any time at his/her own request. Reasons for discontinuation from the study may include the following:

- Refused further follow-up;
- Lost to follow-up;
- Death;
- Study terminated by sponsor;
- AEs;
- Participant request;
- Investigator request;
- Protocol deviation.
-

If a participant does not return for a scheduled visit, every effort should be made to contact the participant. All attempts to contact the participant and information received during contact attempts must be documented in the participant's source document. In any circumstance, every effort should be made to document participant outcome, if possible. The investigator or their designee should capture the reason for withdrawal in the CRF for all participants.

If a participant withdraws from the study, he/she may request destruction of any remaining samples taken and not tested, and the investigator must document any such requests in the site study records and notify the sponsor accordingly.

If the participant withdraws from the study and also withdraws consent for disclosure of future information, no further evaluations should be performed and no additional data should be collected. Any data collected before such withdrawal of consent may continue to be used.

6.18.2 Withdrawal of Consent

Participants should notify the investigator in writing of the decision to withdraw consent from future follow-up, whenever possible. Reasons for withdrawal of consent should be explored as thoroughly as possible and explained in detail in the medical records by the investigator, as to whether the withdrawal is only from further receipt of study intervention or also from study procedures and/or posttreatment study follow-up and entered on the appropriate CRF page.

6.18.3 Loss to Follow-up

A participant will be considered lost to follow-up if he or she repeatedly fails to return for scheduled visits and is unable to be contacted by the study site. The following actions must be taken if a participant fails to attend a required study visit:

- The site must attempt to contact the participant and reschedule the missed visit as soon as possible and ascertain whether the participant wishes to and/or should continue in the study.
- Before a participant is deemed lost to follow-up, the investigator or designee must make every effort to regain contact with the participant (where possible, 3 telephone calls or equivalent methods). These contact attempts should be documented in the participant's study record.

7.0 SAFETY ASSESSMENTS

7.1 Adverse Events

An adverse event is defined as any untoward medical occurrence in a clinical investigation participant administered a pharmaceutical product, which does not necessarily have a causal relationship with this treatment. An adverse event can therefore be any unfavourable and/or unintended sign (including an abnormal laboratory finding), symptom, or disease temporally associated with the use of an investigational medicinal product, whether or not related to the investigational medicinal product. All adverse events, including observed or volunteered problems, complaints, or symptoms, are to be recorded on the appropriate eCRF. This will include the date and time of onset, a description of the AE, severity, duration, actions taken, outcome and an investigator's current opinion on the relationship between the vaccine and the event. A diagnosis and final opinion on the relationship between the study vaccine and the event will be provided at the end of the study by the Investigator.

Wherever possible, a specific disease or syndrome rather than individual associated signs and symptoms should be identified by the Investigator and recorded on the eCRF. However, if an observed or reported sign or symptom is not considered a component of a specific disease or syndrome by the Investigator, it should be recorded as a separate adverse event on the eCRF. Additionally, the condition that led to a medical or surgical procedure (e.g., surgery, endoscopy, tooth extraction, or transfusion) should be recorded as an adverse event, not the procedure.

Any medical condition already present at study enrolment should not be reported as an adverse event unless the medical condition or signs or symptoms present at Screening change in severity or seriousness at any time during the study. In this case, it should be reported as an adverse event.

7.2 Assessment of Unsolicited Adverse Events by the Investigator

The Investigator will assess the severity (intensity) of each adverse event as mild, moderate, severe, or potentially life-threatening and will also categorize each adverse event as to its potential relationship to study vaccine using the categories of not related, unlikely, possible, or probable.

Assessment of Severity: Grade 1-4

- Grade 1, Mild – An event that is easily tolerated and not interfering with normal daily activities.
- Grade 2, Moderate – An event sufficiently discomforting to interfere with normal daily activities.
- Grade 3, Severe – An event associated with inability to work or perform normal daily activities.
- Grade 4, Potentially life-threatening

Causality Assessment:

The relationship of an adverse event to the administration of the vaccine is to be assessed according to the following definitions:

- Not related – The event is clearly related to other factors such as the participant's clinical state, therapeutic interventions, or concomitant drugs administered to the participant. This is especially so when an event occurs prior to receipt of vaccine.
- Unlikely – The event was most likely produced by other factors such as the participant's clinical state, therapeutic interventions, or a concomitant drug administered to the participant and does not follow a known response to the vaccine.
- Possible – The event follows a reasonable temporal sequence from the time of administration of vaccine or follows a known response to the vaccine but could have been produced by other factors such as the participant's clinical state, other therapeutic interventions, or concomitant drugs administered to the participant.
- Probable – The event follows a reasonable temporal sequence from the time of administration of vaccine and follows a known response to the vaccine and cannot be reasonably explained

by other factors such as the participant's clinical state, other therapeutic interventions, or concomitant drugs administered to the participant.

The following factors should also be considered:

- The temporal sequence from vaccine administration
  - The event should occur after the vaccine is given. The length of time from vaccine exposure to event should be evaluated in the clinical context of the event.
- Underlying, concomitant, intercurrent diseases
  - Each report should be evaluated in the context of the natural history and course of the disease being treated and any other disease the participant may have.
- Concomitant drug
  - Other drugs the participant is taking or treatment the participant receives examined to determine whether any might be recognized to cause the event in question.
- Known response pattern for this class of vaccine
  - Based on clinical data for MMR vaccine in this age group
- Exposure to physical and/or mental stresses
  - Exposure to stress sufficient to provide a logical and better explanation for the event.

7.3 Safety Monitoring

Professor Michael Tatley, MB ChB(CapeTown) FFCM(SA), Director of the New Zealand Pharmacovigilance Centre, including Centre for Adverse Reaction Monitoring (CARM) has agreed to be the medical monitor for this study. Safety monitoring will be conducted by the principal investigator and the medical monitor.

7.4 Serious Adverse Events

An adverse event or adverse reaction is considered serious if, in the view of either the Investigator or the Sponsor or designee, it results in any of the following outcomes:

- Death;
- A life-threatening adverse event;
  - NOTE: An adverse event or adverse reaction is considered "life-threatening" if, in view of either the Investigator or Sponsor or designee, its occurrence places the participant at immediate risk of death. It does not include an event that, had it occurred in a more severe form, might have caused death.
- Requires hospitalization or prolongation of existing hospitalizations;
  - NOTE: Any hospital admission with at least one overnight stay will be considered an inpatient hospitalization. An emergency room visit without hospital admission will not be recorded as a SAE under this criterion, nor will hospitalization for a procedure scheduled or planned before signing of informed consent. However, unexpected complications and/or prolongation of hospitalization that occur during elective surgery should be recorded as adverse events and assessed for seriousness. Admission to the hospital for social or situational reasons (i.e., no place to stay, live too far away to come for hospital visits) will not be considered inpatient hospitalizations.
- A persistent or significant disability/incapacity or substantial disruption of the ability to conduct normal life functions;
- A congenital anomaly/birth defect; or

- An important medical event.
    - NOTE: Important medical events that may not result in death, be life-threatening, or require hospitalization may be considered an SAE when, based upon appropriate medical judgment, they may jeopardize the participant and may require medical or surgical intervention to prevent one of the outcomes listed above. Examples of such medical events include allergic bronchospasm requiring intensive treatment in an emergency room or at home, blood dyscrasias or convulsions that do not result in inpatient hospitalizations, spontaneous abortion/miscarriage, or the development of drug dependency.

7.5 Serious Adverse Event Reporting − Procedures for Investigators

Initial Reports

All SAEs occurring from the time of signing the informed consent form until the end of the study must be reported to the sponsor within 24 hours of the knowledge of the occurrence (this refers to any adverse event that meets any of the aforementioned serious criteria). All SAEs that the Investigator considers related to vaccine occurring after the study period must be reported to the Sponsor or designee.

To report the SAE, complete the SAE form in the electronic data capture (EDC) system for the study. When the form is completed, the sponsor will be notified electronically and will retrieve the form. In addition, email the sponsor to notify them of the SAE report. If the event meets serious criteria and it is not possible to access the EDC system, send an email to the sponsor within 24 hours of awareness. When the EDC system becomes available, the SAE information must be entered within 24 hours of the system becoming available.

In addition, the Investigator should complete an online Adverse Event Following Immunisation report to the NZ Pharmacovigilence Centre for Adverse Reactions Monitoring (https://nzphvc.otago.ac.nz/report/).

Follow-Up Reports

The Investigator must continue to follow the participant until the SAE has subsided or until the condition becomes chronic in nature, stabilizes (in the case of persistent impairment), or the participant dies.

Within 24 hours of receipt of follow-up information, the Investigator must update the SAE form electronically in the EDC system for the study and submit any supporting documentation (e.g., participant discharge summary or autopsy reports) to the sponsor via or e-mail. If it is not possible to access the EDC system, refer to the procedures outlined above for initial reporting of SAEs.

7.6 Pregnancy Reporting

Participants with pregnancies detected during the study will be referred for registration or enrolment into pregnancy registries run by the vaccine manufacturer or the Ministry of Health.

7.7 Expedited Reporting

The Sponsor or designee will report all relevant information about suspected unexpected serious adverse reactions that are fatal or life-threatening as soon as possible to the NZ Pharmacovigilance Centre for Adverse Reactions Monitoring and, in any case, no later than 7 days after knowledge by Sponsor or designee of such a case, and that relevant follow-up information will subsequently be communicated within an additional 8 days.

All other suspected unexpected serious adverse reactions will be reported to the NZ Pharmacovigilance Centre for Adverse Reactions Monitoring as soon as possible but within a maximum of 7 days of first knowledge by Sponsor or designee.

7.8 Safety Review Committee

A Safety Review Committee (SRC) has been appointed for this study with members Professor Michael Tatley and Dr Jennifer Lee. Both have expertise in assessment of vaccine safety. A Charter has been prepared for the Safety Review Committee (see Appendix 2).

8.0 IMMUNOLOGY ASSESSMENTS

8.1 Antibody responses

The primary immunological endpoint for this study is measles IgG as measured before and after receipt of Priorix MMR vaccine using the Micro Immune Assay (MIA) at RIVM, Netherlands. The MIA assay is multiplex, also measuring antibody to mumps, rubella and varicella antigens. Mumps and varicella antibody also measured by Southern Community Laboratories, Dunedin prior to MMR vaccine receipt and measles antibody before and after MMR vaccine receipt. Participants whose MIA antibody to measles is below 0.30 units will also be measured using a Plaque Reduction Neutralising Titre (PRNT) assay at RIVM.

8.2 **Measles vaccine virus in oral fluid specimens**

Oral fluid specimens collected using the Oracol device for collecting gingival fluid will be selectively tested for the presence of measles vaccine virus (MVV) by PCR at RIVM. Samples from participants with a fourfold or greater antibody rise from baseline will be prioritised for testing, with the aim of determining if MVV replication has occurred contemporaneously with marked antibody rise in comparison with control specimens where no antibody rise has been detected.

8.3 **Return of results**

Study participants will be provided with an individual level summary of their results when these have been returned and re-identified. Their results will also be reported back to Student Health Services, to ensure vaccination requirements have been complied with.

9.0 DATA ANALYSIS AND STATISTICAL METHODS

9.1 Sample Size

The target sample size for each vaccine cohort (aerosol, intradermal, intramuscular administration) is 100 persons.

9.2 Determination of Sample Size

The sample size was based on estimates from past years that among health science students screened for measles and mumps antibody at the University of Otago students, about 250 will require MMR, around 150 of whom will have measles antibody below threshold. If 60% consent to participate in the randomised study, this will be 50 per arm (aerosol, intradermal, intramuscular) in year 1 and 50 per arm in year 2, a total of 100 per arm across two years. Of the remaining 100 students required to have MMR, we anticipate that at least 70% or 70 per year will agree to allow the study to have access to their pre vaccine screening blood sample and post vaccine blood sample, or 140 across 2 years. Based on deeming a 15% difference (77% vs 92%) in vaccine response clinically important, we require 87 participants per group to detect a difference at least this large with alpha=0.05 and power=80%. Allowing for 10% drop-out, this becomes 97 participants per group, within our anticipated recruitment target. We will evaluate recruitment and seroconversion in year 1 and take measures to increase recruitment if necessary for year two.

9.3 General Statistical Methods

Data from both study years will be analysed. The number and percentage of participants screened, enrolled, vaccinated and completing follow-up will be summarized. Summary descriptive statistics will be presented for the presenting demographic characteristics, including ethnicity and clinical features by study group and subgroups. These will include means, medians, standard deviation and ranges for continuous measures and frequencies and percentage for categorical measures.

Safety data including all solicited local and systemic AEs (reactogenicty) occurring within 7 days after each vaccination, unsolicited AEs within 28 days after each vaccination, medically-attended and serious adverse events (SAEs) will be summarised as frequencies and percentages for each study group. The incidence estimates will be included with exact 95% confidence intervals. All MedDRA coded AEs will also be summarised by relationship to vaccination and severity grade. Vaccine acceptability will be assessed after completing vaccination regimen, including willingness to receive vaccine regimen again or recommend to friends/family members.

Immunogenicity results will be summarised as proportions reaching threshold values deemed to represent protection against clinically typical measles (0.12 MIA units, 12 IU by PRNT) and proportions achieving a two or fourfold increase from baseline for each route of vaccine administration. Similarly, Geometric mean titre (GMT) values with 95% confidence intervals will be calculated for each route of vaccine administration at baseline and compared between time points post MMR (days 7, 14, 28 and at 12 months). Statistical significance will be considered to be reached when the difference has an associated P value of < 0.05.

CONFIDENTIAL

10.0 DATA MANAGEMENT AND RECORD KEEPING

10.1 **Method of Participant Identifier Assignment**

- Participants will be identified on all eCRFs by a unique reference number.
- eCRFs are confidential documents and will only be available to the Sponsor or designee, the investigator, and the biostatistician for this study. The principal clinical investigator will maintain, as part of the investigation file, a list identifying all participants entered into the trial.

10.2 **Reporting and Recording of Data**

An electronic eCRF database will be developed for this study and all data will be entered according to the instructions provided in the eCRF Completion Guidelines and ICH/GCP Guidelines. eCRFs must be completed for each participant who has provided written informed consent and received a dose of MMR vaccine by any route. Information including, but not limited to, birth year and the reason the participant was not enrolled will be collected on potential participants who withdraw consent prior to dosing.

Study participants will be identified by a participant ID number. Participant names or other personal identification must be blacked out on any documents submitted to the Sponsor or their designee, with participant ID number and protocol number transcribed onto each document page.

All eCRF data must be reviewed by the principal investigator. The principal investigator will sign off upon completion of all eCRFs for a participant. The Sponsor and/or its designee will verify the data entered into the eCRFs against applicable source documents to ensure accuracy and completeness of the data.

For data entered directly into eCRFs, the eCRF is source. If paper source (such as a signed paper informed consent form) or separate electronic source (such as an electronic medical record) are used, then the paper or other electronic documentation must be retained and made available for monitoring. Study data in this protocol will be “source verified” only if paper source transcription or electronic source transcription precede eCRF data entry. To comply with the requirement to maintain accurate case histories, the Investigator should review and electronically sign the completed eCRF for each participant before the database is locked.

10.3 **Medical Information Coding**

For medical information, the following thesauri will be used:

- Latest version of MedDRA for medical history and adverse events, and
- World Health Organization Drug Dictionary for prior and concomitant medications.

10.4 **Data Validation**

Validation checks programmed within the EDC system, as well as supplemental validation performed via review of the downloaded data, will be applied to the data to ensure accurate, consistent, and reliable data. Data identified as erroneous, or data that are missing, will be referred to the investigative site for resolution through data queries.

The eCRFs must be reviewed and electronically signed by the Investigator or designee.

11.0 INVESTIGATOR REQUIREMENTS AND QUALITY CONTROL

11.1 Regulatory Compliance

The investigator will ensure that this study is conducted in full compliance with the protocol, the Declaration of Helsinki, ICH GCP guidelines and all other applicable local laws and regulations. Compliance with these standards provides assurance that the rights, safety, and well-being of participants are protected. The investigator and all research staff participating in this study are expected to adhere to this protocol, applicable privacy laws, and any approval requirements imposed by the EC.

At all times, the study will comply with the Aotearoa New Zealand Health and Disability Commissioner's Code of Health and Disability Services Consumers' Rights and Regulations.

The sponsor (or designee)/investigator will obtain, from the clinical sites' EC prospective approval of the clinical protocol and corresponding informed consent form(s); modifications to the clinical protocol and corresponding informed consent forms, and advertisements (i.e., directed at potential research participants) for study recruitment.

The clinical site(s) EC will operate in compliance applicable International Conference on Harmonization (ICH) Guidelines on Good Clinical Practice (GCP), and any other pertinent individual country laws/regulations.

11.2 Ethics Committee (EC) Approval

This study will be conducted in compliance with the Declaration of Helsinki and its amendments and the applicable regulations within New Zealand.

The Health and Disability Ethics (HDEC) committee, New Zealand, must review and fully approve the ethical components of the study, the investigator's informed consent document, and related participant information and recruitment materials before the start of the study.

11.3 Study Initiation

Before the start of this study at each study site, the following documents must be on file:

- All regulatory documentation as required by local and national regulations (completed by the investigator with the assistance of the Sponsor or designee).
- Current curricula vitae of the principal investigator and all sub-investigators.
- Written documentation of EC approval of ethic aspects of protocol and informed consent form.
- A copy of the HDEC-approved informed consent document. Site-specific informed consent documents must be reviewed and approved by the Sponsor or designee prior to use.
- Documentation of site personnel training.
- A signed investigator agreement.

11.4 Informed Consent

Informed consent shall be obtained in writing and documented before a participant is enrolled in the clinical investigation in accordance with the terms and definitions in accordance with ICH GCP.

It is the responsibility of the investigator to ensure that written informed consent is obtained from the participant before any activity or procedure is undertaken and the process of obtaining informed consent is documented.

CONFIDENTIAL

11.5 Amendments and Deviations

11.5.1 Protocol Amendments

This protocol is to be followed exactly. Changes to the research covered by this protocol must be implemented by formal protocol amendment. Amendments to the protocol may be initiated by the Sponsor or designee or at the request of the investigator. In either case, a formal amendment cannot be initiated until the principal investigator has signed it off, and it has been approved by the EC.

11.5.2 Emergency Deviations

Emergency deviations or modifications may be initiated only in cases where change is necessary to eliminate an immediate apparent hazard to participants. Emergency deviations or modifications must be reported to the medical monitor, principal investigator and the EC no later than 24 hours after the emergency.

11.5.3 Protocol Deviations

Deviation from the clinical protocol and protocol requirements including ICH/GCP guidelines must be reported to the Sponsor or designee in a timely fashion. All protocol deviations will be reviewed and evaluated on an ongoing basis and appropriate corrective actions implemented as necessary.

12.0 STUDY ADMINISTRATIVE INFORMATION

12.1 Study Monitoring Requirements

The standard procedures for monitoring will be followed per Good Clinical Practice (GCP) and the study protocol. It is the investigator's responsibility to ensure accurate completion of the eCRFs and to approve the eCRFs.

12.2 Case Report Forms

The study will use an electronic data capture system. All eCRFs will be designed and provided to the site by the sponsor or designee. All personnel accessing the electronic data capture system will be trained on the use of the system by the Sponsor or designee responsible for data management.

eCRFs will be completed for each participant screened for the clinical study. Clinical study data recorded directly in the eCRF, whereupon the eCRF data is to be considered the source data, is acceptable.

Participant names will not be supplied to the Sponsor. A participant ID number will be recorded in the eCRF. Study findings stored on a computer will be stored in accordance with local data protection laws. The participants will be informed that representatives of the EC or regulatory authorities may inspect their medical records to verify the information collected, and that all personal information made available for inspection will be handled in strictest confidence and in accordance with local data protection laws. Electronic systems will be used as the sole instrument for the recording and analysis of clinical and laboratory data related to the safety.

12.3 Data Management

Record handling and processing will be per GCP and the data and tissue management plan.

12.4 Disclosure of Data

Participant medical information obtained for this study is confidential, and disclosure to third parties other than those noted below is prohibited. Participant data will be identified by study, participant ID number and birth year. No other personal identifiers will be used, and data will be de-identified in a manner compliant with local regulations.

Medical information may be given to the participant's personal physician or other appropriate medical personnel responsible for their welfare.

Data generated by this study will be used by the Sponsor or designee in connection with the ongoing evaluation of the vaccines being studied and thus must be available for inspection upon request by representatives of national and local health authorities and the EC for each study site.

12.5 Retention of Records

The Sponsor requires that records and documents pertaining to the conduct of this study and the distribution of investigational drug, including eCRFs, consent form, dosing records, and laboratory test results be retained by the principal investigator for a period of 15 years after the study has been completed. Records should not be destroyed unless agreed to by the sponsor in writing.

12.6 Data Quality Assurance

Data from the study will be entered into eCRFs in a validated Redcap database. Data review, coding, and logic, range, cross-form, and consistency checks will be performed to ensure quality of the data. Adverse events and medications will be coded using MedDRA and the World Health Organization Drug Dictionary (WHO-DD).

12.7 Publications

Study report(s) will be prepared in accordance with regulations and will be provided to the ECs as required. The Sponsor or designee intends to submit the results of this investigation for publication as conference proceeding(s) and/or medical journal article(s) upon completion of the study. This will be preceded by feedback to participants and feedback as lay summaries to community representatives including Māori through a hui process Study investigators may be invited by the Sponsor or designee to contribute. After the initial study data have been published, additional data may be published by the study investigators.

12.8 SCOTT Assessment

An application was made to the New Zealand Medicines and Medical Devices Safety Authority (MEDSAFE), via the Standing Committee on Therapeutic Trials (SCOTT).
SCOTT/Medsafe approval for the trial was deemed not required as Priorix MMR vaccine is an approved medicine in New Zealand.

CONFIDENTIAL

13.0 REFERENCES

1. Cherry JD, Zahn M Clinical characteristics of measles in previously vaccinated and unvaccinated patients in California Clin Inf Diseases 2018; 67:1315-1319
2. Griffin DE The immune response in measles: virus control, clearance and protective immunity Viruses 2016; 8:282; doi:10.3390/v8100282
3. Moss W Measles in vaccinated individuals and the future of measles elimination Clin Inf Diseases 2019; 67:1320-1321
4. Gibney KB, Attwood LO, Nicholson S, Tran T, Druce J, Healy J et al Emergence of Attenuated Measles Illness Among IgG-positive/IgM-negative Measles Cases: Victoria, Australia, 2008-2017. Clin Infect Dis 2020; 70:1060-1067doi: 10.1093/cid/ciz363
5. ESR weekly surveillance report for measles Week 51: 14-20 December 2019 https://surv.esr.cri.nz/PDF_surveillance/MeaslesRpt/2019/WeeklyMeasles09232019.pdf
6. Sá Machado R, Perez Duque M, Almeida S, et al. Measles outbreak in a tertiary level hospital, Porto, Portugal, 2018: challenges in the post-elimination era. *Euro Surveill*. 2018; 23: 18-00224. doi:10.2807/1560-7917.ES.2018.23.20.18-00224
7. Bolotin S, Hughes SL, Gul N, et al. What Is the Evidence to Support a Correlate of Protection for Measles? A Systematic Review. *J Infect Dis*. 2020; 221:1576-1583. doi:10.1093/infdis/jiz380
8. Chen RT, Markowitz LE, Albrecht P, Stewart JA, Mofenson LM, Preblud SR, Orenstein WA. Measles antibody: reevaluation of protective titers. J Infect Dis. 1990; 162:1036-42. doi: 10.1093/infdis/162.5.1036
9. Hahné SJ, Nic Lochlainn LM, van Burgel ND, Kerkhof J, Sane J, Yap KB, van Binnendijk RS. Measles Outbreak Among Previously Immunized Healthcare Workers, the Netherlands, 2014. J Infect Dis. 2016; 214: 1980-1986. doi: 10.1093/infdis/jiw480
10. Rosen JB, Rota JS, Hickman CJ, Sowers SB, Mercader S, Rota PA et al Outbreak of measles among persons with prior evidence of immunity, New York City, 2011. Clin Infect Dis 2014; 58:1205-10. doi: 10.1093/cid/ciu105.
11. Woudenberg T, van Binnendijk R, Veldhuijzen I, Woonink F, Ruijs H, van der Klis F, Kerkhof J, de Melker H, de Swart R, Hahné S. Additional Evidence on Serological Correlates of Protection against Measles: An Observational Cohort Study among Once Vaccinated Children Exposed to Measles. Vaccines 2019; 7:158. doi: 10.3390/vaccines7040158
12. de Swart RL, de Vries RD, Rennick LJ, et al. Needle-free delivery of measles virus vaccine to the lower respiratory tract of non-human primates elicits optimal immunity and protection. *NPJ Vaccines*. 2017; 2:22 doi:10.1038/s41541-017-0022-8
13. Fiebelkorn AP, Coleman LA, Belongia EA, Freeman SK, York D, Daoling B et al Measles virus neutralising antibody response, cell-mediated immunity, and Immunoglobulin G antibody avidity before and after receipt of a third dose of measles, mumps, rubella vaccine in young adults J Inf Diseases 2016; 213: 1115-1123
14. Kaaijk P, Wijmenga-Monsuur AJ, van Houten MA, Veldhuijzen IK, Ten Hulscher HI, Kerkhof J, van der Klis FR, van Binnendijk RS. A Third Dose of Measles-Mumps-Rubella Vaccine to Improve Immunity Against Mumps in Young Adults. J Infect Dis. 2020; 221:902-909 doi: 10.1093/infdis/jiz188
15. McLean HQ, Fiebelkorn AP, Ogee-Nwankwo A, Hao L, Coleman LA, Adebayo A, Icenogle JP. Rubella virus neutralizing antibody response after a third dose of measles-mumps-rubella vaccine in young adults. Vaccine. 2018; 36: 5732-5737. doi: 10.1016/j.vaccine.2018.08.010
16. Bianchi FP, Stefanizzi P, De Nitto S, Larocca AMV, Germinario C, Tafuri S. Long-term Immunogenicity of Measles Vaccine: An Italian Retrospective Cohort Study. *J Infect Dis*. 2020; 221:721-728. doi:10.1093/infdis/jiz508
17. Griffin DE. Current progress in pulmonary delivery of measles vaccine. *Expert Rev Vaccines*. 2014; 13:751-759. doi:10.1586/14760584.2014.915753
18. Dilraj A, Cutts FT, de Castro JF, Wheeler JG, Brown D, Roth C, Coovadia HM, Bennett JV. Response to different measles vaccine strains given by aerosol and subcutaneous routes to schoolchildren: a randomised trial. Lancet 2000; 355: 798-803 doi: 10.1016/s0140-6736(99)95140-1
19. Diaz-Ortega J-L, Bennett JV, Castaneda D, Arellano DM, Martinez D, de Castro JF. Safety and Antibody Responses to Aerosolized MMRII Vaccine in Adults: An Exploratory Study World

CONFIDENTIAL

Journal of Vaccines 2012; 2:55-60
20. Low N, Bavdekar A, Jeyaseelan L, et al. A randomized, controlled trial of an aerosolized vaccine against measles. *N Engl J Med*. 2015; 372: 1519-1529 doi:10.1056/NEJMoa1407417
21. Low N, Kraemer S, Schneider M, Restrepo AM. Immunogenicity and safety of aerosolized measles vaccine: systematic review and meta-analysis. Vaccine 2008 17;383-98. doi: 10.1016/j.vaccine.2007.11.010
22. Diaz-Ortega JL, Bennett JV, Castaneda D, Martinez D, Fernandez de Castro J. Aerosolized MMR vaccine: evaluating potential transmission of components to vaccine administrators and contacts of vaccinees. Biologicals. 2012; 40:278-81 doi: 10.1016/j.biologicals.2012.03.002.
23. Cohen BJ, Parry RP, Andrews N, Bennett AM, Dennis JH. Laboratory methods for assessing vaccine potency retained in aerosol outputs from nebulizers: application to World Health Organization measles aerosol project. Vaccine 2008; 26:3534-9 doi: 10.1016/j.vaccine.2008
24. Fink JB, Ehrmann S, Li J, Dailey P et al Reducing Aerosol-Related Risk of Transmission in the Era of COVID-19: An Interim Guidance Endorsed by the International Society of Aerosols in Medicine. J Aerosol Med Pulm Drug Deliv. 2020 Aug 12. doi: 10.1089/jamp.2020.1615
25. Beals CR, Railkar RA, Schaeffer AK, et al. Immune response and reactogenicity of intradermal administration versus subcutaneous administration of varicella-zoster virus vaccine: an exploratory, randomised, partly blinded trial. *Lancet Infect Dis*. 2016; 16: 915-922. doi:10.1016/S1473-3099(16)00133-X
26. Prausnitz MR, Goodson JL, Rota PA, Orenstein WA. A microneedle patch for measles and rubella vaccination: a game changer for achieving elimination *Curr Opin Virol*. 2020; 41:68-76. doi:10.1016/j.coviro.2020.05.005
27. Latner DR, Sowers SB, Anthony K, Colley H, Badeau C, Coates J et al Qualitative Variation among Commercial Immunoassays for Detection of Measles-Specific IgG. J Clin Microbiol. 2020; 58: e00265-20. doi: 10.1128/JCM.00265-20
28. Dorigo-Zetsma JW, Leverstein-van Hall MA et al including van Binnendijk RS. Immune status of health care workers to measles virus: evaluation of protective titers in four measles IgG EIAs. J Clin Virol. 2015; 69:214-8 doi: 10.1016/j.jcv.2015.06.095
29. Vyse AJ, Cohen BJ, Ramsay ME. A comparison of oral fluid collection devices for use in the surveillance of virus diseases in children. Public Health 2001; 115: 201-7 doi: 10.1038/sj/ph/1900751 7
30. Hutse V, Van Hecke K, De Bruyn R, et al. Oral fluid for the serological and molecular diagnosis of measles. *Int J Infect Dis*. 2010;14: e991-e997. doi:10.1016/j.ijid.2010.06.009
31. Measles Aerosol Vaccine Project – Report to SAGE 2012 https://www.who.int/immunization/sage/meetings/2012/november/3_MeaslesAerosolVaccineProject-report_SAGE.pdf?ua=1 accessed November 15th 2020

14.0 APPENDICES

14.1 Appendix 1. Abbreviations

AE	Adverse event
AESI	Adverse event of special interest
CRF	Case report from
eCRF	Electronic case report form
EC	Ethic committee
EDC	Electronic data capture
GCP	Good clinical practices
GMT	Geometric mean titre
ICH	International Conference on Harmonization
IEC	Institutional ethics committee
IRB	Institutional Review Board
MAAE	Medically-attended adverse event
MedDRA	Medical Dictionary for Regulatory Authority
SRC	Safety Review Committee
SOP	Standard operating procedure
SOA	Schedule of assessments

14.2 **Appendix 2. Safety Monitoring Committee**

Safety Monitoring Committee – Roles and responsibilities

Safety Monitoring Committee: Role

The role of the Safety Monitoring Committee (SMC) is to provide expert, timely and independent review of data collected to monitor safety during the conduct of the clinical trial "Measuring and boosting waning immunity to measles in young adults."

Safety Monitoring Committee: Responsibilities

1. Review data obtained for safety assessments
    - Record of participant responses to yes/no questions on adverse events via text prompts at 12, 24, 48 and 72 hours and specifics of any "Yes" responses
    - Data on swelling, redness and reported pain at injection site at visit 2 (3-4 days), visit 3 (7-8 days), visit 4 (14-16 days)
    - Data on systemic symptoms reported by participants at visit 2 (3-4 days), visit 3 (7-8 days), visit 4 (14-16 days)

2. Review any clinical data that may be relevant to this review (e.g. demographics, vaccination timing, medications)

3. Be notified of any events meeting criteria for Serious Adverse Events (SAEs) within 24 hours of the Principal Investigator becoming aware of their occurrence
    - Evaluate causality criteria for any reported SAEs

4. Ensure recommendations are based upon an unbiased and comprehensive evaluation of the data.

Safety Monitoring Committee: Independence

It is essential that the judgment of the SMC members is not influenced by factors other than those necessary to maintain subject safety and to preserve the integrity of the trial. The medically qualified professionals who have agreed to be members of the SMC do not have any involvement with the clinical trial conduct or reporting and will not be authors on publications or otherwise stand to benefit from SMC membership.
