# Supplementary material for: Immunogenicity and safety of measles-mumps-rubella vaccine delivered by the aerosol, intradermal and intramuscular routes in previously vaccinated young adults: a randomized controlled trial protocol
Source: PLoS One. 2025 Mar 21;20(3):e0318893. doi: 10.1371/journal.pone.0318893 (PMC11927902; doi:10.1371/journal.pone.0318893)
Supplement: Attachment — Submitted filename: Response_to_Reviewers_auresp_2.docx [file pone.0318893.s008.docx]

Dear Reviewers,

Thank you for your feedback. Based on your comments, we have amended the manuscript further and tweaked a few sentences to enhance clarity. Along with this document, we have uploaded a clean manuscript and a version with tracked changes (in colored text). Please find below the pointwise responses to your comments.

Thank you.

| **Reviewers’ comment** | **Authors’ reply** |
| --- | --- |
| **Reviewer #1:** | |
| The authors have adequately addressed the comments. I have no further comments. The manuscript is suitable for publication. | Thank you for your feedback. |
| **Reviewer #2:** | |
| 1. L21 the word ‘re-introduction’ is not appropriate. | Done.  The revised sentence reads as the following-  ‘There are increasing reports of outbreaks of measles in countries that had eliminated the infection using two doses of measles-mumps-rubella (MMR) vaccine.’ |
| 2. Check the use or not of ‘the’ before ‘measles vaccine’ in L29, L31, and L54, respectively and all over the manuscript. | Done.  We checked and updated it in a few instances where it appeared appropriate. |
| 3. Check the plural form in L25 ‘administration’, L96-97 ‘aerosol and intradermal delivery’, L107 ‘delivery’, and L108 and L125 ‘antibody’. | Done.  We checked and updated the plural forms as advised. |
| 4. L51-52, L94, and in the entire manuscript, what do the authors mean by ‘settings’? | Settings is a general word commonly used by the World Health Organization (WHO) and other authors to designate situations and places, including countries.  E.g.,  *1. Bolotin, Shelly et al. “In Elimination Settings, Measles Antibodies Wane After Vaccination but Not After Infection: A Systematic Review and Meta-Analysis.” The Journal of infectious diseases vol. 226,7 (2022): 1127-1139. doi:10.1093/infdis/jiac039*  *2. Measles outbreaks strategic response plan 2021–2023. Geneva: World Health Organization; 2021. (https://www.who.int/publications/i/item/9789240018600)* |
| 5. This sentence needs to be revised: ‘As measles remain endemic in different parts of the globe, individual countries where elimination was achieved following prolonged periods of high two-dose measles vaccination coverage and very low measles incidence have increasingly documented measles infection in people with two documented measles vaccine doses. For example, 26/232 (11%) cases in US outbreaks (California; 2000-2015), (8) 6/32 (19%) cases in an Australian outbreak (New South Wales 2012), (9) and 11/94 (11.7%) cases during a South Korean outbreak in 2019. (10)’ | Done.  The updated sentence reads as the following:  ‘Measles remains endemic in many countries. As a result, countries where elimination has been achieved following prolonged periods of high two-dose measles vaccination coverage accompanied by very low measles incidence, when outbreaks occur due to imported cases, have increasingly documented measles infection in people with two documented measles vaccine doses. Examples include US outbreaks where 26/232 (11%) cases in California (2000-2015),[8] 6/32 (19%) cases in an Australian outbreak (New South Wales 2012),[9] and 11/94 (11.7%) cases during a South Korean outbreak in 2019.[10]’ |
| 6. Similarly, add data related to previous measles outbreaks in New Zealand. As the study is being conducted in New Zealand, this would give an overview of past measles infections in that country and the necessity to conduct the current study. The authors can refer to S2 File, especially in the Section ‘Importance’. | Done.  The following sentence has been added to address it:  ‘Similarly, in a large outbreak of over 2100 cases in New Zealand in 2019, about 10% of cases in young adults occurred in two-dose MMR recipients.[11]’  Please note that reference 11 is a new one added to the manuscript.  Reference:  [11] Institute of Environmental Science and Research. Measles weekly reports - 2020, https://www.esr.cri.nz/digital-library/measles-weekly-reports-2020/ (2020, accessed 21 December 2024). |
| 7. L84-89 This sentence needs to be re-checked. | Done.  The updated sentence reads as the following:  ‘VMNs generate 3-5 µm size particles that deposit the vaccine deep into the lung tissue when inhaled.[19] Other than the study cited above,[16] previous studies in adults on the safety and immunogenicity of measles-containing vaccines (MCV) by route of administration have compared subcutaneous administration with aerosol delivery using the Classical Mexican Device for aerosol generation, used vaccine strains not used in high-income elimination settings (such as Edmonston Zagreb (measles) and Leningrad Zagreb or Rubini (mumps strains)), and have predominantly included seropositive subjects.[20, 21]’ |
| 8. L60 ‘As measles remain’, L94 ‘measles is’, and in the entire manuscript: in what context(s) this word is used so that the authors use plural or singular forms after it? | L60 has been amended as below:  ‘Measles remains endemic in many countries. As a result, countries where elimination has been achieved following prolonged periods of high two-dose measles vaccination coverage accompanied by very low measles incidence, when outbreaks occur due to imported cases, have increasingly documented measles infection in people with two documented measles vaccine doses.’  L94 has been amended as below:  ‘Given evidence that measles cases have been increasingly recognised in measles elimination settings in two-dose vaccinated young adults in childhood, whether the strength and/or duration of protection can be improved is a key question.’ |
| 9. L112-113, write entirely ‘December’ like you wrote entirely ‘February’, and not ‘Dec’. | Done.  The updated sentence reads as the following:  ‘The trial started recruiting in February 2023 and will end in December 2025.’ |
| 10. The use or not of ‘the’ before some nouns/group of nouns should be checked in the entire manuscript. | Please see reply to comment no. 2. |
