# Supplementary material for: Immunogenicity and safety of measles-mumps-rubella vaccine delivered by the aerosol, intradermal and intramuscular routes in previously vaccinated young adults: a randomized controlled trial protocol
Source: PLoS One. 2025 Mar 21;20(3):e0318893. doi: 10.1371/journal.pone.0318893 (PMC11927902; doi:10.1371/journal.pone.0318893)
Supplement: Attachment — Submitted filename: Response to Reviewers.docx [file pone.0318893.s007.docx]

Dear Editors,

We thank you for the thorough review of our manuscript and detailed feedback. Below, we have tabulated a pointwise response to your comments and amended a few sentences to ensure contextuality and flow. Along with this document, we have uploaded a clean manuscript and a version with tracked changes (in colored text).

Thank you.

| **No.** | **Reviewer Comments** | **Authors’ reply** |
| --- | --- | --- |
| REVIEWER 1 COMMENTS | | |
| 1. | The following require revision:  177-178 | Following was the sentence in line 177-178: ‘As vaccine potency can vary depending on lot,[33] we will make every attempt to use the same lot unless faced with situations that are beyond the control of the research team, such as supply shortage, vaccine vials crossing expiry date before vaccination day, damage of vaccine potency (e.g., cold chain failure), etc.’  We have updated the above sentence as below-  ‘Though using the same vaccine lot would be ideal, it would not be feasible as our study will be conducted at different geographic locations over a prolonged period (up to 3 years). However, the vaccine lots will be recorded, and antibody responses by vaccine lot will be examined.’ |
| 2. | The following require revision:  191-192 | Following was the sentence in line 191-192: ‘All vaccinators will hold a current authorized vaccinator certificate and will prepare and administer intradermal and aerosol vaccinations utilizing specific training videos and manufacturer instructions.’  We have updated the above sentence as below:  ‘All trial participants will be vaccinated by trained vaccinators equipped for vaccination by the intradermal and aerosol route through familiarity with manufacturer instructions and training videos for the specific devices used.’ |
| 3. | The following require revision:  Page 10. The sentence ‘Redness and swelling are graded as absent, mild (grade 1), moderate (grade 2), or severe (grade 3) based on characteristics categorized as >2-5 cm (mild), >5-10 cm (moderate), and >10 cm (severe).’ | The revised sentence is below:  ‘Redness and swelling are graded as absent, mild (grade 1; >2-5 cm), moderate (grade 2; >5-10 cm), or severe (grade 3; >10 cm).’ |
| 4. | The following require revision:  Page 11, the sentence ‘principal investigator or the sponsor or designee, it results in outcomes’ | The revised sentence is below:  ‘The principal investigator or the sponsor or designee will categorize a side effect as serious if it leads to one of the following: a life-threatening event that puts a participant at immediate risk of death and hospitalization.’ |
| 5. | Page 10, state if $125 in NZD or USD currency. | We have clarified in the manuscript that payments are in New Zealand dollars. |
| 6. | Page 10, typo 'laboratory (RIVM) The principal’ | Corrected. |
| 7. | Page 11, 2023-24 to be written as 2023-2024 | Corrected. |
| 8. | For sample size calculation, detailed information, calculation, the comparison groups for the outcome variables are to be provided. Among the three comparison groups (intramuscular, aerosol, and intradermal), which comparison sample size was used for representation and state whether formula or software/sample size calculator was used to derive the sample size. | Following sample size calculation description is from the submitted version:  ‘Over the two years 2023-24, we anticipate ~1400 students will be screened for eligibility. To compare vaccine delivery methods, we assumed that a difference of ≥15% in seroconversion is clinically significant. To detect this difference in seroconversion with 80% power and a 5% significance level, we need 87 participant-students with 1:1 allocation ratio. With three arms (intramuscular, aerosol, and intradermal), and allowing for 10% dropout, we have a target of 300 students. We anticipate that we can achieve our recruitment target of 300 participants (100 per arm) in our target cohort over two years and will make appropriate adjustments to study sites and recruitment strategy if the review of year 1 results indicates this is necessary.’  We amended the description of sample size estimation as below to include requested additional information:  ‘Over the two years 2023-2024, we anticipate ~1400 students will be screened for eligibility. When comparing efficacy of aerosol delivery and intradermal delivery to intramuscular delivery, we assumed that a difference of ≥15% in seroconversion is clinically significant. To detect this difference in seroconversion with 80% power and a 2-sided 5% significance level, we need 87 participant-students in each group (PASS 2023 software, NCSS, LLC, Kaysville, Utah, USA). With three arms (intramuscular, aerosol, and intradermal), and allowing for 10% dropout, we aim to recruit 100 students per group (300 students in total). We anticipate that we can achieve this recruitment from our target cohort over two years and will make appropriate adjustments to study sites and recruitment strategy if the review of year 1 results indicates this is necessary’ |
| 9. | Page 11, the section Analysis is to be stated as Statistical Analyses. | We updated it in the manuscript. |
| 10. | Page 11, concentrations (GMC) for antibody levels are usually presented as geometric mean with the geometric standard deviation (GSD) or with confidence intervals. | Thanks for pointing it out. Although we mentioned how we will analyze the continuous variables in general, we added the following sentence under the ‘Statistical analysis’ section with respect to GMC to clarify it:  ‘The geometric mean concentrations (GMC) for antibody levels will be reported in mean and 95% confidence intervals.’ |
| 11. | Page 12, ‘chi-square test statistic’ to be written as ‘chi-square test’. | We updated it in the manuscript. |
| 12. | Page 12, the sentence ‘changes from baseline at these two post-vaccination time points...’ requires revision. | The sentence in the submitted version was: ‘The determinants of GMC changes from baseline at these two post-vaccination time points will be determined using univariate and multivariate linear regression models.’  The revised sentence is below:  ‘The predictors of post-vaccination GMC changes will be estimated using linear regression models.’ |
| 13. | Page 12, the sentence ‘The following variables will be considered as candidate covariates in models:’ to be written as ‘The following variables will be considered as candidate covariates in the models’ | The revised sentence is below:  ‘For the regression models, we will determine a predictor-outcome association for the following covariates: sex, age, ethnicity, country of birth, smoking, alcohol intake, history of allergy, medication use, co-morbidities, co-administration of any other vaccine, intervention arm allocation, and baseline antibody titer.’ |
| 14. | Page 12, the sentence ‘While we aim to perform the above analyses in an intention-to-treat manner; given the trial's long-term multiple post-intervention follow-up requirements, participant attrition is possible. Therefore, the primary analysis will follow a per-protocol assessment.’ is unclear and requires revision. | The revised sentence is below:  ‘Participant attrition is anticipated due to the prolonged trial duration and the multiple study visit requirements for biological sample donation. Therefore, the analysis will only include the data available at different time points (per protocol analysis). The missing data will be reported in frequency and percentages and discussed narratively.’ |
| 15. | Subtitle interim analysis, analysis population, addressing missing data and analysis software are to be omitted and the content is to be combined. | We have omitted the subtitles and combined the content. |
| 16. | The one or two-tailed p-value is to be stated. | We stated it in the sample size description (mentioned above; Reviewer 1, comment 8). |
| 17. | Page 13, for the statement 'in secure locations for ten years’’ states the location e.g. server etc. | We will be keeping the data in secure web servers. The revised sentence is below:  ‘The research team (the principal investigator and designated individuals) will retain participant data (in de-identified form) in secure locations (web servers) for ten years following project completion.’ |
| 18. | The list of references did not conform to the journal’s format. | We have updated the formatting of the references following the journal guidelines. |
| 19. | Supplementary files S1-S5 were not available in the PDF version. | While uploading the revised version, we have uploaded supplementary documents in pdf version. If there is any difficulty in accessing these documents, please let us know. |
| REVIEWER 2 COMMENTS | | |
| 20. | The authors may reconsider the following sentence << Only necessary data for specimen identification will be sent to the testing laboratory (RIVM) The principal investigator and designated members will monitor data entry at three monthly intervals and decide on any amendments needed (safety monitoring stated below)>> as follows:  a) << Only necessary data for specimen identification will be sent to the testing laboratory (RIVM). The principal investigator and designated members will monitor data entry at three monthly intervals and decide on any amendments needed (safety monitoring stated below)>>,  b) or << Only necessary data for specimen identification will be sent to the testing laboratory (RIVM), and the principal investigator and designated members will monitor data entry at three monthly intervals and decide on any amendments needed (safety monitoring stated below)>> | Thanks for the suggestions. We opted for the second edit you suggested and updated it in the revised manuscript. |
| 21. | The authors should mention injection sites for intramuscular and intradermal delivery. | Thanks for letting us know. Given the frequent use of intramuscular routes to inject various intramuscular vaccines (e.g., MMR, hepatitis B, Tetanus-Diphtheria vaccine), we assumed it common knowledge and didn’t mention it. However, following your advice, we have briefly mentioned the intramuscular vaccine delivery site in the following manner:  ‘After reconstituting the vaccine in 0.5 ml of diluent, the vaccine will be injected at a 90-degree angle in the outer part of the upper arm using a 23–25-gauge needle to deliver the vaccine deep into the deltoid muscle.’  Regarding the intradermal vaccine delivery, we have already mentioned the vaccine delivery site. For your reference, we are copying it below from the manuscript:  ‘Intradermal administration of vaccine uses the Micronjet600TM device (NanoPass Technologies Ltd, Israel), a device consisting of three 600 µm long microneedles (https://www.nanopass.com/). PRIORIX MMR vaccine is reconstituted to a total volume of 0.3 mL of sterile water and drawn up into a 2mL syringe. After attachment to the syringe, the microneedle is inserted into the participant’s skin in the deltoid region at a 45-degree angle in accordance with manufacturer instructions, and the vaccine dose is manually delivered to the intradermal region. A visible bleb indicates successful intradermal delivery.’ |
| 22. | At the beginning of the ‘’Introduction section’’ (L47-64), could the authors give more information about measles causative agent (definition, mode of replication, and transmission), the manifestation of the disease (clinical symptoms) and provide data of previous measles outbreaks following secondary-dose failure? | We included the following sentences to mention measles definition, mode of replication, transmission, and clinical symptoms:  ‘Measles is a highly contagious infectious disease caused by the measles virus, a negative-sense single-stranded RNA virus of the Morbillivirus genus and Paramyxoviridae family.(1) It has an incubation period of 10-14 days. On entering the epithelial cells of the upper airway of a susceptible host, the virus first infects the myeloid cells (e.g., macrophages and dendritic cells),(2,3) then it migrates to infect regional lymphoid cells and infects T- and B-cells, which eventually enter the bloodstream and infect the epithelial cells of the skin and submucosa of the respiratory tract. The latter results in coryzal symptoms and cough with spread of infectious viral particles via respiratory droplets and small aerosols to other individuals.(4,5) Measles causes a severe systemic disease clinically characterized by symptoms including fever, cough, coryza, conjunctivitis, and rash, but in high income settings rarely mortality.(1)’  We included the following sentences to report data on measles occurrence in two doses of measles containing vaccine recipients during previous outbreaks has been added as below:  ‘As measles remain endemic in different parts of the globe, individual countries where elimination was achieved following prolonged periods of high two-dose measles vaccination coverage and very low measles incidence have increasingly documented measles infection in people with two documented measles vaccine doses. For example, 26/232 (11%) cases in US outbreaks (California; 2000-2015),(8) 6/32 (19%) cases in an Australian outbreak (New South Wales 2012),(9) and 11/94 (11.7%) cases during a South Korean outbreak in 2019.(10)’ |
| 23. | Receipt of ‘’two doses of MMR vaccine’’ and have ‘’measles IgG antibody levels which are negative or equivocal on commercial assays’’ are two important inclusion criteria for this study protocol. We suggest that participants without any proof of written records be excluded from the study (L15). | Much appreciated suggestion. However, we would respectfully like to keep participants who signed a declaration in the trial due to the reasons stated below:  In the absence of a participant’s vaccination record, an ideal way of verifying the receipt of second dose of measles vaccine would be from a national immunization registry (after obtaining required ethical approval); however, historically, New Zealand didn’t have such national register of childhood immunizations until 2005. Since we are recruiting individuals aged ≥18 (as per the eligibility criteria), the youngest enrolled in the first trial year (2023) would have been born on or before 2005. This means that verifying self-declared receipt of 2nd dose of MMR will not be possible from written records. In light of this, we decided to accept individuals who had signed a declaration to vouch for their childhood receipt of two doses of MMR vaccine in the absence of written records as eligible. This is one of the limitations of our trial, which we will acknowledge it while reporting our trial findings and also examine results among those with and without written records. |
| 24. | The study will compare aerosol and intradermal delivery of MMR vaccine with the traditional intramuscular route. The authors only described aerosol and intradermal delivery procedures in <> but omitted to provide details as well for intramuscular delivery although the latter is a traditional route. | Intramuscular delivery is now included in the revised manuscript. |
| 25. | L192 Are the specific training videos for intervention providers already made? | These training videos come from the manufacturers.  The website links to such videos are shared here:   - For aerosol delivery: <https://www.aerogen.com/products/aerogen-solo> - For intradermal delivery: <https://www.nanopass.com/micronjet-microneedle-device/instructions-for-use/> |
| 26. | L205 Why the oral fluid collection will only be made during the first three follow-up visits within two weeks post- vaccination and not until follow-up 5 like venous blood collection (L195)? | The purpose of oral fluid collection in the study is to examine whether measles vaccine virus (MVV) can be detected – which we assume would indicate that sterilizing immunity to measles infection was not present. There are limited data about the prevalence of detection of MVV post vaccination and the data which are available relate to children receiving the vaccine for the first time. We will not collect oral fluid specimens after the first two weeks because based on measles pathophysiology post respiratory tract infection, detection of MVV after two weeks in previously vaccinated individuals in whom it was not detected in the first two weeks would not be expected as reviewed by the report below.  Ref: McMahon J, Mackay IM, Lambert SB. Measles vaccine virus RNA in children more than 100 days after vaccination. Viruses. 2019 Jul 10;11(7):636s |
| 27. | L211-212 <>. Did the authors plan to add another technique to confirm their RT-PCR results? If yes, would the authors like to describe the latter? | We will base our methodology for detection of measles vaccine virus PCR on the methods described in the reference cited above  Ref: McMahon J, Mackay IM, Lambert SB. Measles vaccine virus RNA in children more than 100 days after vaccination. Viruses. 2019 Jul 10;11(7):636 |
| 28. | Would the authors like to specify whether or not there is a control group participant (no vaccine administration)? | While acknowledging that a placebo arm would have been of interest for examination of possible vaccine-related adverse events, we considered that as all participants are required by the University policy to receive MMR vaccine if not seropositive at screening, adding this arm would make recruitment substantially more difficult and conduct of the study substantially more demanding of time and resources. Thus we elected to consider the intramuscular arm as the comparison group as this is standard practice and the relevant standard of care. |
